# Optimization of Water–Nitrogen–Salinity Management for Improving Yield, Quality, and Resource Use Efficiency of Pigment Pepper Under Brackish Water Irrigation in Arid Regions

**DOI:** 10.3390/plants15172573

**Published:** 2026-08-24

**Authors:** Xi Yang, Yao Guan, Xinghong He, Jiaxin Sun, Xiaozhe Liu, Yongrui Pang

**Affiliations:** 1 School of Water Conservancy and Architectural Engineering, Tarim University, Alar 843300, China; 10757242300@stumail.taru.edu.cn (X.Y.); 10757242308@stumail.taru.edu.cn (J.S.); l10757241168@163.com (X.L.); 10757242290@stumail.taru.edu.cn (Y.P.); 2 South Xinjiang Geotechnical Engineering Research Center, Tarim University, Alar 843300, China

**Keywords:** pigment pepper, brackish water irrigation, water–nitrogen–salinity interactions, water use efficiency, nitrogen partial factor productivity, Cloud–TOPSIS

## Abstract

Brackish water utilization provides an alternative strategy for alleviating freshwater scarcity in arid agricultural regions; however, the synergistic regulation of salinity, irrigation, and nitrogen management remains unclear. A two-year field experiment was conducted in 2025 and 2026 to investigate the effects of water–nitrogen–salinity interactions on growth, yield formation, resource use efficiency, and fruit quality of pigment pepper (*Capsicum annuum* L.) under arid conditions in Xinjiang, China. An L9(3^3^) orthogonal experimental design was adopted with three levels of brackish water salinity, irrigation amount, and nitrogen application rate. The comprehensive production performance of different management strategies was further evaluated using a combined weighting Cloud–TOPSIS approach. The results showed that water–nitrogen–salinity interactions significantly regulated pigment pepper growth, yield formation, and resource utilization, with consistent responses observed across the two experimental years. Increasing irrigation water salinity reduced leaf chlorophyll content (CHL) and nitrogen balance index (NBI), whereas flavonoid content (FLAV) exhibited an increasing trend under moderate salinity stress. Low-salinity irrigation combined with appropriate water and nitrogen inputs maintained higher photosynthetic capacity and nitrogen nutritional status. Yield, water use efficiency (WUE), and partial factor productivity of nitrogen (PFPN) were jointly affected by salinity, irrigation, and nitrogen supply. Excessive salinity significantly reduced crop productivity, while optimized irrigation and nitrogen management alleviated salt stress effects. The T2 treatment (1 g L^−1^ salinity, 2400 m^3^ ha^−1^ irrigation, and 300 kg ha^−1^ nitrogen application) achieved the highest yield and maintained favorable WUE and PFPN values in both years. Fruit quality responses demonstrated that moderate salinity promoted capsaicinoid accumulation, whereas excessive salinity restricted biomass production and quality improvement. Correlation analysis revealed that photosynthetic nitrogen metabolism indicators were closely associated with yield formation, while flavonoid accumulation showed stronger relationships with quality attributes. The Cloud–TOPSIS evaluation identified T2 as the optimal management strategy under the experimental conditions by balancing yield, quality, and resource use efficiency. These findings indicate that coordinated regulation of irrigation water salinity, water supply, and nitrogen input is essential for achieving efficient brackish water utilization and sustainable pigment pepper production in arid regions.

## 1. Introduction

Pigment pepper (*Capsicum annuum* L.) is an economically important crop with extensive industrial applications. Extracts derived from its fruits are widely used as natural colorants in the food industry and as raw materials in the pharmaceutical and chemical industries [1]. Xinjiang is characterized by abundant solar radiation, large diurnal temperature variations, and a dry climate, which provide favorable conditions for the growth and development of pigment pepper as well as for the accumulation of pigments, sugars, and other compounds in the fruits. Consequently, Xinjiang has become one of the major pigment pepper-producing regions in China [2,3]. However, as a typical arid and semiarid region, Xinjiang receives limited precipitation and experiences high evaporative demand, and its agricultural production has long been constrained by freshwater scarcity [4]. Against the backdrop of global climate change and intensifying competition for water resources, improving agricultural water use efficiency and optimizing irrigation management strategies have become critical priorities for achieving sustainable agricultural development in arid regions [5]. Therefore, improving the use efficiency of limited water and fertilizer resources while maintaining crop yield and quality is essential for achieving sustainable agricultural development in this region.

Brackish water represents a potential alternative water source and has considerable value for alleviating pressure on freshwater supplies for agricultural irrigation [6]. The long-term application of brackish water may alter the salt environment in the root zone through the continuous input of salt ions, thereby affecting crop water uptake, nutrient utilization, and physiological metabolism. Recent studies have demonstrated that salt stress can further restrict crop growth and yield formation by disrupting plant water balance, ion homeostasis, and photosynthetic processes [7]. Brackish water irrigation may therefore exert contrasting effects on crop performance. Moderate salinity may stimulate osmotic adjustment and promote the accumulation of quality-related compounds, whereas excessive salinity may inhibit plant growth and yield formation [8]. Previous studies have demonstrated that the rational use of brackish water can partially meet the irrigation requirements of salt-tolerant crops. For example, long-term saline water irrigation did not significantly reduce winter wheat yield when irrigation water salinity remained below a certain threshold [9]. Irrigation with low-salinity water has also been reported to improve cotton fiber quality and sunflower seed quality [10,11,12,13]. In addition, Alkalai-Tuvia et al. [14] found that irrigation with moderately saline water enhanced both the external appearance and internal quality attributes of pepper fruits. Nevertheless, most previous studies have focused on a single salinity level or on crop responses to salt stress, and the interactive effects of brackish water salinity, irrigation amount, and nitrogen supply remain insufficiently understood.

Integrated water and fertilizer management has been widely adopted as an important strategy for regulating crop production across diverse agroecosystems. Optimizing the coordination between irrigation and nitrogen application can enhance nutrient uptake, improve water and nitrogen use efficiency, and minimize resource losses caused by excessive agricultural inputs [15]. Appropriate combinations of irrigation and fertilizer inputs can improve plant nutritional status, enhance photosynthetic capacity, and promote yield formation. For example, Wu et al. [16] reported that an appropriate water and fertilizer supply significantly enhanced photosynthesis, yield, and water use efficiency in tomato. However, in arid and salt-affected regions, water availability, nutrient inputs, and soil salinity do not act independently; rather, they interactively regulate crop performance by jointly modifying the root-zone environment and plant physiological processes. In recent years, brackish water has emerged as a promising alternative irrigation water source in arid and semi-arid regions. However, its successful application largely depends on the salinity level of irrigation water, irrigation management practices, and the salt tolerance of the cultivated crop [17]. Therefore, single-factor optimization based solely on irrigation or fertilizer application cannot adequately capture the coordinated effects of water, nutrients, and salinity under actual field conditions.

In recent years, multi-criteria comprehensive evaluation methods have been increasingly applied to the optimization of agricultural management practices. Integrating indicators of growth performance, yield, resource use efficiency, and fruit quality enables a more comprehensive assessment of the overall production performance under different management strategies [18]. In recent years, multi-criteria evaluation approaches have been increasingly applied to agricultural management decision-making, facilitating the optimization of agricultural production systems by simultaneously considering productivity, resource use efficiency, and environmental impacts [19]. For example, Li et al. used principal component analysis to evaluate the overall performance of different tomato germplasm resources, while Mehmood et al. [20] applied the Technique for Order Preference by Similarity to an Ideal Solution (TOPSIS) to optimize irrigation scheduling, thereby simultaneously increasing yield and reducing resource consumption. Nevertheless, current research on pigment pepper production in arid regions has largely focused on the effects of individual environmental factors on yield or fruit quality. The combined effects of irrigation, nitrogen application, and salinity under brackish water conditions remain insufficiently understood, and few studies have comprehensively evaluated management strategies by simultaneously considering yield formation, resource use efficiency, and fruit quality.

Therefore, a fixed-site field experiment was conducted using pigment pepper grown in the arid region of Xinjiang to investigate different combinations of brackish water salinity, irrigation level, and nitrogen application rate. The effects of these water–nitrogen–salinity combinations on leaf physiological traits, including the chlorophyll index (CHL), flavonol index (FLAV), and nitrogen balance index (NBI), as well as yield formation, water use efficiency (WUE), partial factor productivity of applied nitrogen (PFPN), and fruit quality, were systematically evaluated to characterize the production responses of pigment pepper to the interactive effects of irrigation, nitrogen supply, and salinity. Furthermore, a multi-criteria evaluation framework integrating the cloud model with the Technique for Order Preference by Similarity to an Ideal Solution (cloud–TOPSIS) was employed to identify an optimal water–nitrogen–salinity management strategy that simultaneously achieves high yield, superior fruit quality, and efficient resource use [21]. The findings are expected to provide a theoretical basis and technical support for the safe utilization of brackish water and the resource-efficient production of pigment pepper in the arid region of Xinjiang.

## 2. Materials and Methods

### 2.1. Experimental Site

Field experiments on pigment pepper were conducted during the 2025 and 2026 growing seasons at the Water-Saving Irrigation Experimental Station of the College of Water Resources and Architectural Engineering, Tarim University, Xinjiang, China (40°32′ N, 81°17′ E; 1013 m above sea level). Each experimental plot measured 4.8 m × 10.0 m, and the treatments were arranged in a randomized design.

**Figure 1:**
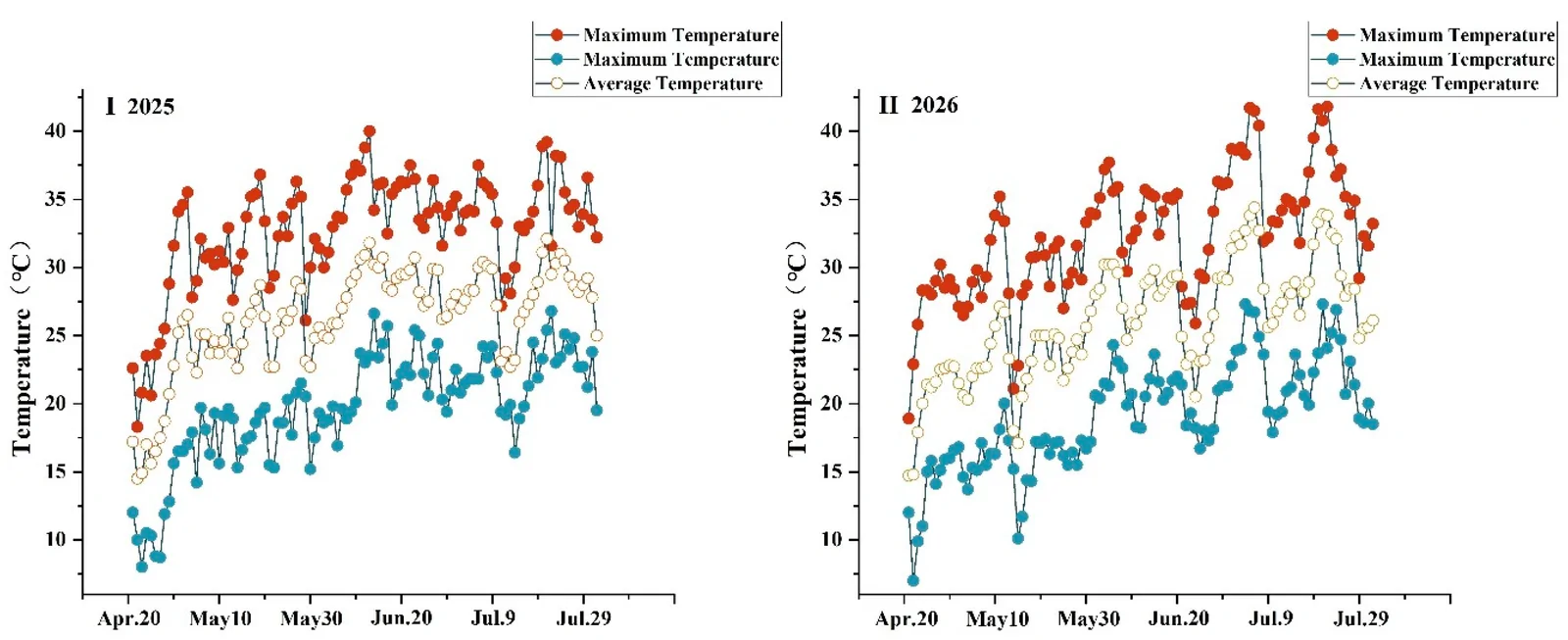
Field temperature changes during the cultivation period in (**I**) 2025 and (**II**) 2026.

The experimental site has a warm-temperate, extremely continental climate characterized by scarce precipitation and intense surface evaporation. From May to July, the mean daily sunshine duration is approximately 9.5 h. The mean annual air temperature is 10.7 °C, and the mean annual precipitation is approximately 50 mm. Figure 1 shows the variations in air temperature during the two experimental growing seasons, and Table 1 lists the basic physicochemical properties of the soil in the two growing seasons. These environmental and soil background data were provided to characterize the comparability of experimental conditions between the two growing seasons and to facilitate interpretation of treatment effects under field conditions.

**Figure 2:**
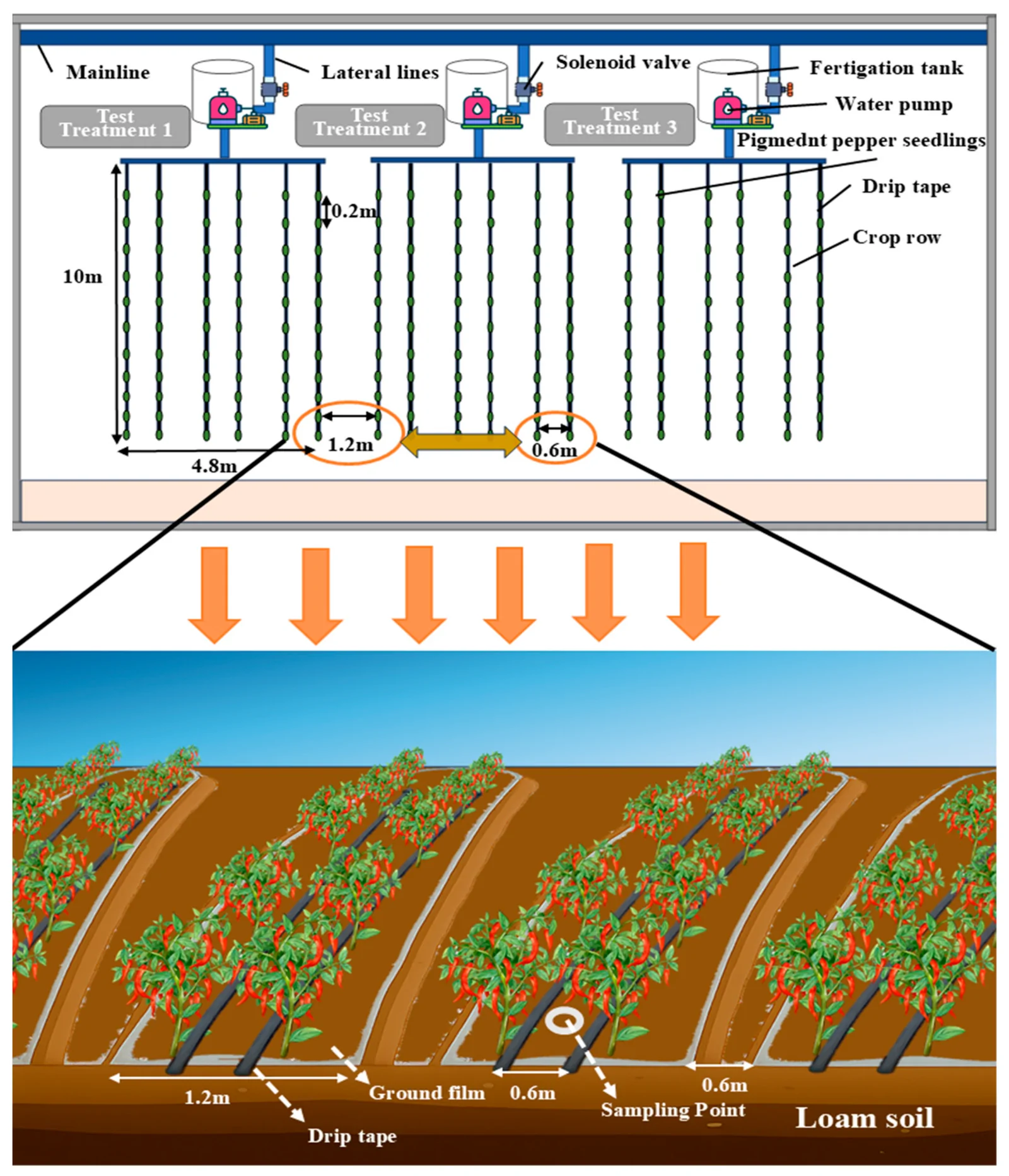
Layout diagram of drip irrigation tape for pigment peppers and schematic diagram of the field experiment design.

The locally cultivated pigment pepper cultivar ‘Honglong 23’ was used in this study. The planting configuration is illustrated in Figure 2. A cultivation pattern consisting of two crop rows and two drip lines under each plastic-mulched bed was adopted. The plastic film was 120 cm wide, with a spacing of 60 cm between adjacent mulched beds. Both the drip-line spacing and row spacing were 60 cm, and the within-row plant spacing was 20 cm. In-line emitter drip tapes were used, with an emitter spacing of 20 cm and a nominal emitter discharge rate of 2.6 L h^−1^.

During land preparation, 300 kg ha^−1^ of an effective microorganism (EM) microbial inoculant(Xinjiang Yinghao Biotechnology Development Co., Ltd., Xinjiang, China) was incorporated into the soil. Urea containing 45% N, monoammonium phosphate containing 70% NH_4_H_2_PO_4_, and potassium sulfate containing 42% K_2_O (Xinjiang Lop Nur Potassium Salt Co., Ltd., Xinjiang, China) were used as the sources of nitrogen, phosphorus, and potassium, respectively.

Considering the regional climatic conditions and the growth cycle of pigment pepper, seedlings were transplanted in late spring in both experimental years. In 2025, transplantation was performed on 1 May. The seedling establishment stage lasted from 1 May to 4 June, followed by the flowering and fruit-setting stage from 4 June to 5 July and the fruit-coloring stage from 5 to 31 July. In 2026, seedlings were transplanted on 25 April. The corresponding growth stages were defined as the seedling establishment stage from 25 April to 24 May, the flowering and fruit-setting stage from 24 May to 30 June, and the fruit-coloring stage from 30 June to 31 July.

### 2.2. Experimental Design and Irrigation–Fertigation Management

The experiment was arranged according to an L_9_(3^3^) orthogonal design (Table 2), in which nine treatment combinations were selected from the full set of 27 possible combinations. Three factors were considered, each at three levels: brackish water salinity (factor A), irrigation amount (factor B), and nitrogen application rate (factor C). Each treatment was replicated three times to ensure balanced representation of the factor levels. An additional control treatment (CK) was irrigated with freshwater (A0) at an irrigation amount of 2400 m^3^ ha^−1^ and a nitrogen application rate of 300 kg ha^−1^. Thus, the experiment comprised a total of 30 plots.

The brackish irrigation water was prepared by dissolving NaHCO_3_, Na_2_SO_4_, NaCl, CaCl_2_, and MgCl_2_ (Xinjiang Salt Lake Salt Manufacturing Co., Ltd., Xinjiang, China) in freshwater at specified proportions to simulate the ionic composition of local natural brackish water. According to the recommended application rates for water-soluble compound fertilizers, all treatments received the same amounts of phosphorus and potassium, at 180 and 270 kg ha^−1^, respectively.

Irrigation and fertilizer were applied through an integrated drip fertigation system. A total of 12 irrigation events were scheduled throughout the growing season at 7-day intervals. Two irrigation events were applied during the seedling establishment stage, accounting for 10% of the seasonal irrigation amount; three during the flowering stage, accounting for 20%; four during the fruit-development stage, accounting for 50%; and three during the fruit-coloring stage, accounting for the remaining 20%.

Fertilizer was applied with each irrigation event, resulting in 12 fertigation events during the growing season. Of the total seasonal fertilizer input, 5% was applied during the seedling establishment stage, 15% during the flowering stage, 60% during the fruit-development stage, and 20% during the fruit-coloring stage.

### 2.3. Measurements

#### 2.3.1. Soil Sampling

Soil samples were collected before transplanting and after completion of the experimental treatments. At each sampling event, soil water content, salinity, and temperature were measured in the 0–20, 20–40, 40–60, and 60–80 cm soil layers using a Zhishang soil sensor (Zhangjiakou Sansheng Smart Agriculture Technology Co., Ltd., Zhangjiakou, China). The collected soil samples were placed in plastic bags and immediately transported to the laboratory. After passing through a 2 mm sieve, the samples were stored at 4 °C until subsequent analyses.

Soil electrical conductivity (EC) was determined using soil samples collected with an auger at a horizontal distance of 5 cm from the drip line. Samples were obtained from the 0–10, 10–20, 20–40, and 40–60 cm soil layers, and the sampling locations are illustrated in Figure 2. The samples were air-dried, gently crushed, and passed through a 2 mm sieve. A soil–water suspension was then prepared at a soil-to-water ratio of 1:5. After shaking for 3 min, the suspension was filtered, and the electrical conductivity of the filtrate was measured to characterize the soil’s soluble salt content in accordance with the Chinese agricultural industry standard NY/T 1121.16–2006.2.

#### 2.3.2. Leaf Physiological Trait Measurements

Beginning at the seedling stage, five uniformly growing pepper plants were randomly selected from each treatment for physiological measurements. Leaf chlorophyll content (CHL), flavonoid content (FLAV), and nitrogen balance index (NBI) were determined at four growth stages: the seedling stage, flowering and fruit-setting stage, full fruiting stage, and fruit color transition stage. For each plot, fully expanded functional leaves located above the second branch were collected from five randomly selected plants and measured using a Dualex polyphenol–chlorophyll meter (Dualex, Force-A, Orsay, France) according to the manufacturer’s instructions [22].

#### 2.3.3. Fruit Biomass and Yield Measurements

At the end of the fruiting period, five representative fruits were randomly collected from each plot to determine individual fruit fresh weight, dry weight, and dry-to-fresh weight ratio.

After sampling, soil particles and other impurities adhering to the fruit surface were gently removed using a soft brush. Surface moisture was then removed with absorbent paper, and the fresh weight of each fruit was measured using an electronic balance with a precision of 0.01 g.

For dry weight determination, the fruit samples were initially heated in a forced-air oven at 105 °C for 30 min to rapidly inactivate enzymatic activity. The samples were subsequently dried at 75 °C for 48 h and cooled in a desiccator for 30 min before weighing. They were then dried for an additional 2–4 h, cooled, and reweighed. This drying and weighing procedure was repeated until the difference between two consecutive measurements was ≤0.002 g. The final constant weight was recorded as fruit dry weight.

The dry-to-fresh weight ratio was calculated as follows:

Dry-to-fresh weight ratio (%) = fruit dry weight/fruit fresh weight × 100.

In addition, five representative plants were randomly selected from each plot, and the total number of fruits per plant was recorded. Dry fruit yield per plant was estimated as the product of fruit number per plant and mean individual fruit dry weight.

#### 2.3.4. Fruit Quality Analysis

At the late fruiting stage, ten fruits were randomly collected from each plot and combined into a composite sample for quality analysis. The contents of capsaicin, dihydrocapsaicin, and vitamin C were determined using high-performance liquid chromatography (HPLC) according to the method described by [23]. Each sample was analyzed in triplicate, and the mean value was used for subsequent analyses.

#### 2.3.5. Water and Nitrogen Use Efficiency

Water use efficiency (WUE) was calculated as the ratio of pigment pepper yield to the total seasonal irrigation amount:

(1) $$WUE=\frac{Y}{IA}$$

where WUE is the water use efficiency (kg m^−3^), (Y) is the fruit yield (kg ha^−1^), and (IA) is the total irrigation water applied during the growing season (m^3^ ha^−1^).

The partial factor productivity of applied nitrogen (PFPN), which represents the crop yield obtained per unit of nitrogen fertilizer applied, was calculated as follows:

(2) $$PFPN=\frac{Y}{F}$$

where PFPN is the partial factor productivity of applied nitrogen (kg kg^−1^), and (F) is the nitrogen application rate (kg N ha^−1^).

### 2.4. Statistical Analysis and Comprehensive Evaluation

Microsoft Excel 2023 (Microsoft Corp., Redmond, WA, USA) was used for data organization and preliminary calculations. Analysis of variance (ANOVA) and range analysis of the orthogonal experiment were performed using IBM SPSS Statistics 23.0 (IBM Corp., Armonk, NY, USA). Brackish water salinity, irrigation amount, and nitrogen application rate were treated as fixed effects, whereas experimental year and replicate were treated as random effects. The Sidak method was used to analyze the effects of two-way and three-way interactions on the measured indicators. Where appropriate, potential covariates were incorporated into the statistical model to account for variation unrelated to the imposed treatments. Differences among treatment means were considered statistically significant at *p* < 0.05.

Figures were prepared using MATLAB 2024 (MathWorks, Natick, MA, USA) and Origin 2024 (OriginLab Corp., Northampton, MA, USA). Based on the results of the multi-criteria orthogonal experiment, the weights of the evaluation indicators were determined using a combined weighting method. An improved cloud model–TOPSIS approach was subsequently applied to comprehensively evaluate the effects of water–nitrogen–salinity management on the overall production performance of pigment pepper and to identify the optimal management strategy.

#### 2.4.1. A Comprehensive Scoring Method for Determining the Weights of Evaluation Factors

Assume that the evaluation system comprises (m) experimental treatments and (n) evaluation indicators. The original decision matrix is defined as X = [$\chi_{\mathrm{ij}}$]. To eliminate the influence of dimensions, range standardization is adopted:

Positive indicators:

(3) $$\gamma_{\mathrm{ij}}=\frac{\chi_{\mathrm{ij}}-\min\left(\chi_{j}\right)}{\max\left(\chi_{j}\right)-\min\left(\chi_{j}\right)}$$

Reverse indicator:

(4) $$\gamma_{\mathrm{ij}}=\frac{\max\left(\chi_{j}\right)-\chi_{\mathrm{ij}}}{\max\left(\chi_{j}\right)-\min\left(\chi_{j}\right)}$$

Obtain the standardized matrix R = [$\gamma_{\mathrm{ij}}$].

To reduce the subjectivity associated with expert-based weighting and the uncertainty caused by data variability, a combined analytic hierarchy process (AHP) and entropy weight method was adopted to determine the weights of evaluation indicators. The AHP method was used to quantify the relative importance of indicators based on expert knowledge and pairwise comparisons, while the entropy weight method was applied to determine objective weights according to the degree of information provided by each indicator.

For the AHP (analytic hierarchy process) method, a pairwise comparison matrix was established based on the relative importance of indicators using a nine-point scale proposed by Saaty. The normalized eigenvector of the matrix was calculated to obtain the subjective weights of each indicator. The consistency ratio (CR) was further calculated to evaluate the reliability of the judgment matrix, and CR values below 0.10 were considered acceptable.

Finally, the combined weights were calculated by integrating the subjective weights obtained from AHP and the objective weights derived from the entropy method. Therefore, we use α and β to represent, respectively, the relative importance degree of the subjective weight and the objective weight. Here, we will use the idea of a matrix to measure the importance coefficients $\alpha_{i}$ and $\beta_{i}$ of the subjective weight and the objective weight, where i=1,2,⋯,n, and the formula is as follows:

(5) $$\alpha_{i}=\frac{\nu_{i}}{\left(\nu_{i}+\omega_{i}\right)}$$

(6) $$\beta_{i}=\frac{\omega_{i}}{\left(\nu_{i}+\omega_{i}\right)}$$

Here, $\nu_{i}$ is the subjective weight obtained by the AHP (analytic hierarchy process), and $\omega_{i}$ is the objective weight obtained by the fusion of the entropy method. After obtaining the importance coefficients $\alpha_{i}$ and $\beta_{i}$ of the subjective weight and the objective weight, we can obtain the comprehensive weight $Q_{i}$ of each indicator. The formula is as follows:

(7) $$Q_{i}=\frac{\left(\nu_{i}\alpha_{i}+\omega_{i}\beta_{i}\right)}{\left(\nu_{i}+\omega_{i}\right)}$$

Substitute the primary and secondary weights obtained from Formulas (5) and (6) into Formula (7) and determine the weight coefficients using the resulting combined weighting method. The processed data and the weight coefficients determined by the combined weighting method are introduced into the Cloud–TOPSIS model for comprehensive evaluation.

#### 2.4.2. The TOPSIS Method Improved Based on the Cloud Model

**Figure 3:**
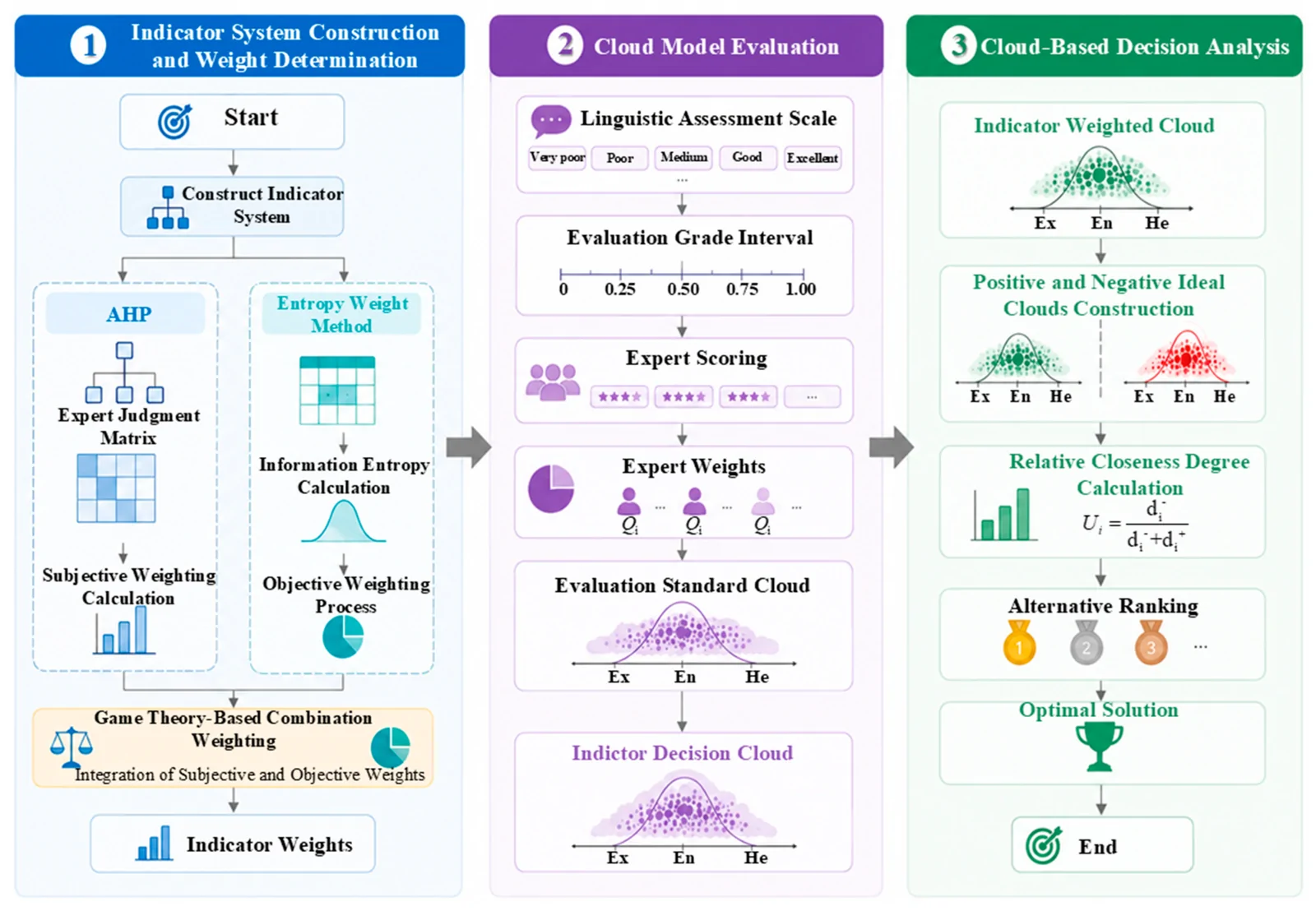
Cloud–TOPSIS evaluation process.

The Cloud–TOPSIS evaluation process is shown in Figure 3. The TOPSIS method evaluates the relative performance of alternative schemes by calculating their distances from the positive and negative ideal solutions. However, in constructing a comprehensive evaluation system for pigment pepper growth and production, the evaluation information may involve both fuzziness and randomness, while the decision-making process may also be influenced by behavioral factors such as preference heterogeneity and reference dependence. To account for these uncertainties, the cloud model was introduced to quantitatively represent qualitative and linguistic evaluation information. The cloud model was then integrated with TOPSIS to construct the positive and negative ideal cloud sets. The relative closeness of each treatment to the ideal cloud sets was calculated to rank and identify the optimal treatment schemes. This integrated approach was used to improve the discriminatory capacity and robustness of the comprehensive evaluation. The detailed procedure was as follows:

1. Positive and negative ideals converge. For each indicator j, select the positive or negative ideal cloud from the indicator-weighted cloud ${\tilde{r}}_{ij}$ of all schemes:
  (8) $$R^{+}=\left\{r_{j}^{+}|\underset{1\leq i\leq m}{\max}r_{ij}\right\}$$
  (9) $$R^{-}=\left\{r_{j}^{-}|\underset{1\leq i\leq m}{\min}r_{ij}\right\}$$
  In this formula, $\max_{i}\left({\tilde{r}}_{ij}\right)$ indicates that the weighted cloud with the largest expected Ex is preferred. If there are multiple cases where the expected value Ex is equal, then choose the one with smaller En and He (with less uncertainty and higher stability). $\min_{i}\left({\tilde{r}}_{ij}\right)$ represents the weighted cloud of the index with the smallest expected Ex. Similarly, first compare the smallest Ex, and if Ex is equal, take the one with smaller En and He.
2. Cloud distance coefficient and distance between alternatives and ideal clouds: The cloud distance coefficient was used to quantify the distance between the weighted cloud and the ideal cloud. Based on the Euclidean distance of the three numerical characteristics of the cloud model, the cloud distance coefficient between the weighted cloud and the ideal cloud was defined as follows:
  (10) $$d({\tilde{r}}_{ij}\;,r_{j}^{\pm })=\sqrt{(\tilde{E}x_{ij}\;-Ex_{j}^{\pm }{)}^{2}+(\tilde{E}n_{ij}\;-En_{j}^{\pm }{)}^{2}+(\tilde{H}e_{ij}-He_{j}^{\pm }{)}^{2}}$$
  Based on this index, the distance coefficient between the weighted cloud and the positive and negative ideal comprehensive cloud is shown in the following formula:
  (11) $$d_{i}^{+}=\sqrt{\sum_{j}^{m}d^{2}(r_{\mathrm{ij}},r_{j}^{+})}$$
  (12) $$d_{i}^{-}=\sqrt{\sum_{j}^{m}d^{2}(r_{\mathrm{ij}},r_{j}^{-})}$$
  where d (r_ij_, r_j_^+^) and d (r_ij_, r_j_^-^) represent the cloud distance coefficients between the weighted cloud of treatment (i) and the positive and negative ideal clouds, respectively.
3. Comprehensive closeness degree and ranking: The comprehensive closeness degree (relative closeness) of treatment (i) was defined as follows:
  (13) $$U_{i}=\frac{d_{i}^{-}}{d_{i}^{-}+d_{i}^{+}}$$
  The larger $U_{i}$ is, the closer the treatment scheme is to the positive ideal cloud and the farther it is from the negative ideal cloud, and the better the comprehensive performance of the pigment pepper. Conversely, it is even worse. Sort each processing plan from largest to smallest according to $U_{i}$ to obtain the optimal processing plan.

## 3. Results

### 3.1. Effects of Water–Nitrogen–Salinity Interactions on Pigment Pepper Growth

**Figure 4:**
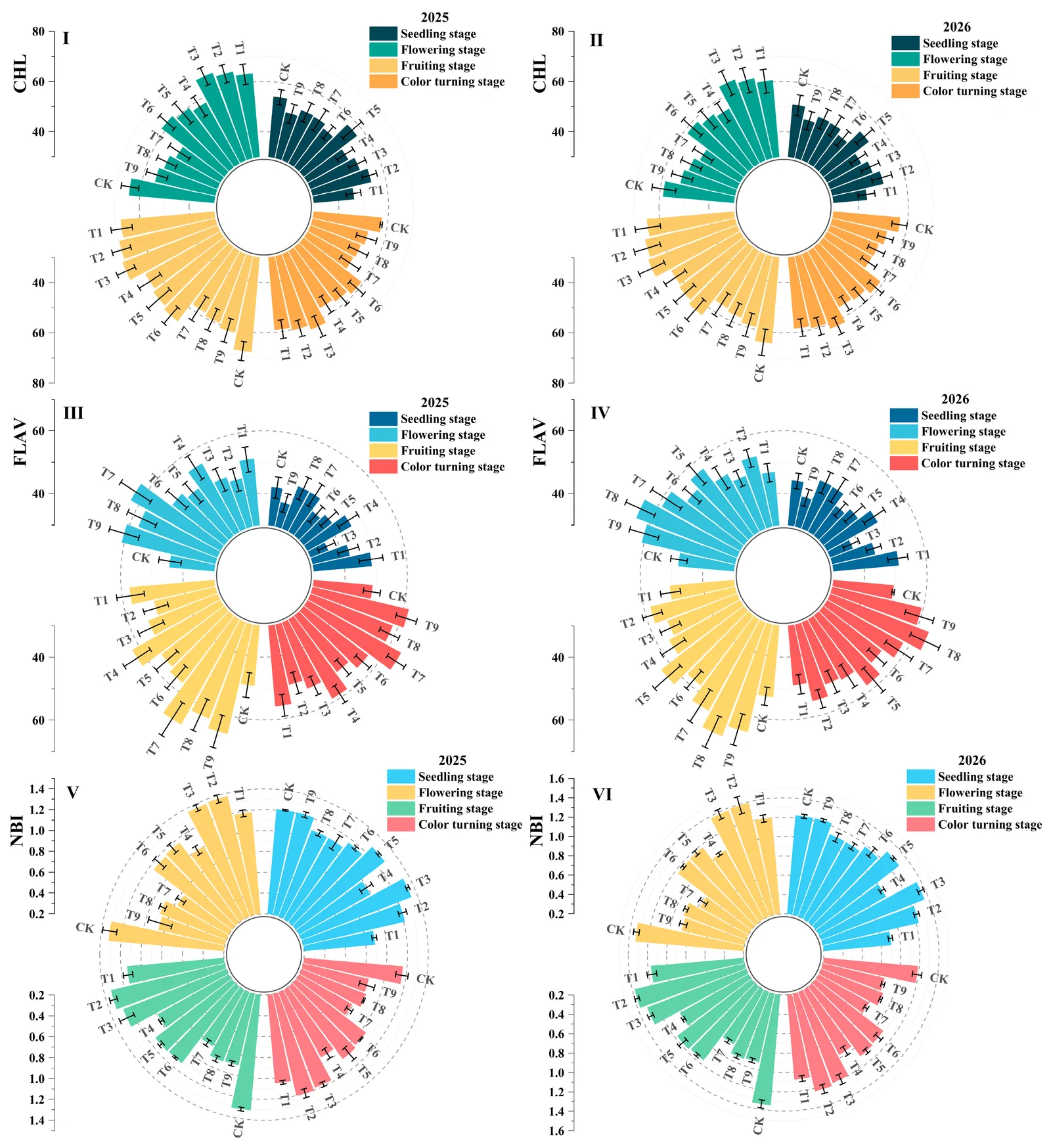
Responses of leaf chlorophyll index (CHL), flavonol index (FLAV), and nitrogen balance index (NBI) of pigment pepper to different water–nitrogen–salinity treatments at different growth stages in 2025 and 2026. Panels (**I**–**VI**) represent the changes in these indicators across growth stages during the two growing seasons.

Water–nitrogen–salinity treatments significantly affected the leaf physiological status of pigment pepper throughout the entire growing season. Distinct variations in leaf chlorophyll index (CHL), flavonol index (FLAV), and nitrogen balance index (NBI) were observed among different combinations of irrigation water salinity, irrigation amount, and nitrogen application rate (Figure 4). Overall, all three indicators exhibited similar temporal variation patterns across the two experimental years, suggesting consistent responses of pigment pepper growth to different water–nitrogen–salinity management regimes. As the growing season progressed, leaf spectral parameters varied dynamically across growth stages, and differences among treatments became increasingly evident. Under low-salinity irrigation conditions, appropriate water and nitrogen supplies maintained a relatively higher leaf nutritional status.

**Figure 5:**
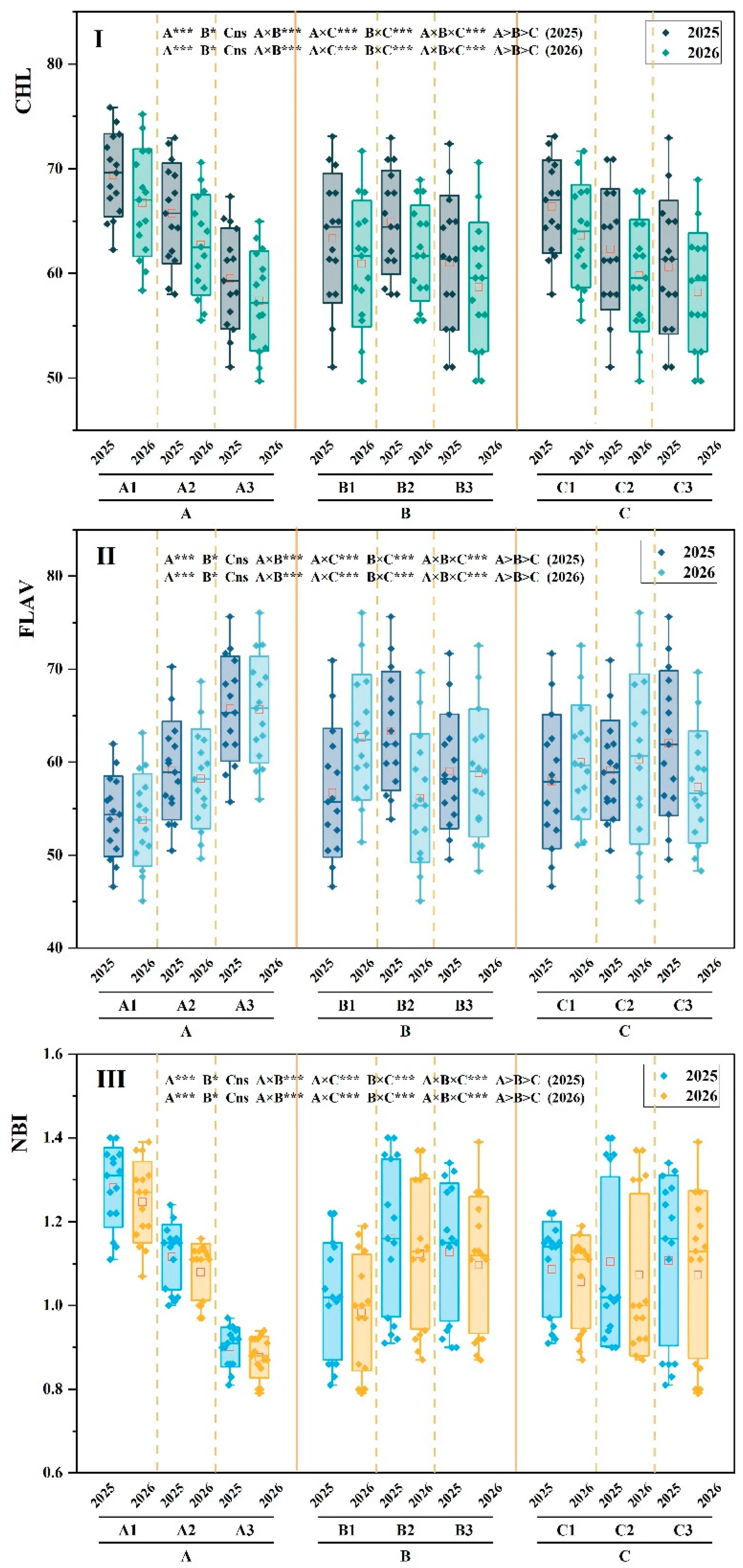
Dynamic changes in leaf optical indices of pigment pepper under different water–nitrogen–salinity treatments across growth stages in 2025 and 2026: (**I**) CHL; (**II**) FLAV; (**III**) NBI. (The box represents the interquartile range (IQR), the horizontal line inside the box indicates the median value, and the whiskers represent the range of data distribution. Points beyond the whiskers indicate outliers). A × B, A × C, B × C, and A × B × C indicate the two-way and three-way interaction effects among salinity (A), irrigation amount (B), and nitrogen application rate (C), respectively, as analyzed by the Sidak method. Significance levels: * (*p* < 0.05), ** (*p* < 0.01), *** (*p* < 0.001), ns (*p* ≥ 0.05).

Different water–nitrogen–salinity treatments significantly affected the leaf chlorophyll index (CHL) of pigment pepper (Figure 5I). Across the two experimental years, CHL exhibited clear dynamic variation patterns at different growth stages. During the seedling establishment stage, CHL values were relatively low in all treatments, with limited differences among treatments. As plants progressed into the flowering and fruit-development stages, leaf CHL gradually increased and reached higher levels during fruit development, indicating enhanced accumulation of photosynthetic pigments during crop growth. During the fruit-coloring stage, CHL decreased in some treatments, suggesting a decline in leaf physiological activity at the late growth stage.

The accumulation of CHL was markedly influenced by different water–nitrogen–salinity combinations. Under low-salinity irrigation conditions (A1), CHL values were generally higher than those under moderate- and high-salinity treatments, and treatment T2 maintained relatively high CHL levels across multiple growth stages. In contrast, high-salinity irrigation (A3) significantly reduced leaf CHL, with more pronounced differences observed during the flowering and fruit-development stages.

Under the same salinity level, variations in irrigation amount and nitrogen application rate further influenced CHL accumulation. Moderate increases in irrigation and nitrogen supply generally enhanced leaf CHL values, whereas excessive or insufficient water and nitrogen inputs did not result in continuous increases in CHL. The results from both experimental years indicated that appropriate water–nitrogen–salinity combinations were beneficial for maintaining higher leaf chlorophyll levels and improving the photosynthetic status of pigment pepper during growth.

The flavonol index (FLAV) reflects the level of secondary metabolism and stress responses in plants. Significant differences in FLAV were observed among different water–nitrogen–salinity treatments (Figure 5II). Across the two experimental years, FLAV generally showed an increasing trend throughout the growth period. During the seedling establishment stage, FLAV values were relatively low in all treatments. With the progression into the flowering and fruit-development stages, FLAV gradually increased with plant growth and metabolic activity and remained at relatively high levels during the fruit-coloring stage.

FLAV exhibited distinct responses to different water–nitrogen–salinity conditions. Under low-salinity irrigation, some water–nitrogen combinations maintained relatively low FLAV values, whereas increasing irrigation water salinity generally promoted FLAV accumulation. Unlike CHL, which declined with increasing salinity, FLAV showed a tendency to accumulate under high-salinity conditions. Among different water and nitrogen combinations, treatments with moderate irrigation amounts and appropriate nitrogen application rates generally maintained a better leaf metabolic status. In contrast, under high-salinity conditions, increasing water and nitrogen inputs did not consistently stabilize FLAV values, and greater fluctuations were observed, indicating that salinity exerted a strong influence on leaf metabolic responses.

The nitrogen balance index (NBI), which reflects leaf nitrogen nutritional status, was significantly affected by different water–nitrogen–salinity treatments (Figure 5III). The two-year results showed similar temporal variation patterns of NBI across growth stages. During the seedling establishment stage, considerable differences in NBI were observed among treatments. As plants entered the vigorous vegetative growth period, NBI gradually increased and reached relatively high levels during the flowering or early fruit-development stages. Subsequently, NBI declined in some treatments with the progression of fruit development.

Comparison among treatments showed that NBI values were generally higher under low-salinity conditions, with treatment T2 exhibiting relatively high NBI values across multiple growth stages, indicating a favorable leaf nitrogen status under this treatment. With increasing salinity, NBI generally decreased, and leaf nitrogen status was negatively affected under high-salinity irrigation. Irrigation amount and nitrogen supply also played important regulatory roles in NBI variation. Under suitable irrigation conditions, increased nitrogen application enhanced NBI values, whereas excessive nitrogen input did not result in a continuous increase in NBI, suggesting that leaf nitrogen status was jointly influenced by water and salinity conditions.

Overall, the two-year results demonstrated that different leaf physiological indices exhibited distinct responses to water–nitrogen–salinity factors. CHL was primarily associated with low-salinity conditions and appropriate water and nitrogen supply, whereas FLAV showed an increasing tendency with increasing salinity. NBI was more sensitive to nitrogen availability and salinity conditions.

Throughout the growth period, the effects of water–nitrogen–salinity interactions on pigment pepper leaf physiological characteristics exhibited clear stage-dependent patterns. Differences among treatments were relatively small during the seedling establishment stage but became increasingly evident during the flowering and fruit-development stages. Treatment T2 consistently maintained relatively high CHL and NBI values across multiple growth stages while sustaining an appropriate level of FLAV accumulation, indicating superior leaf physiological performance.

### 3.2. Effects of Water–Nitrogen–Salinity Interactions on Yield Formation and Water–Fertilizer Use Efficiency

**Figure 6:**
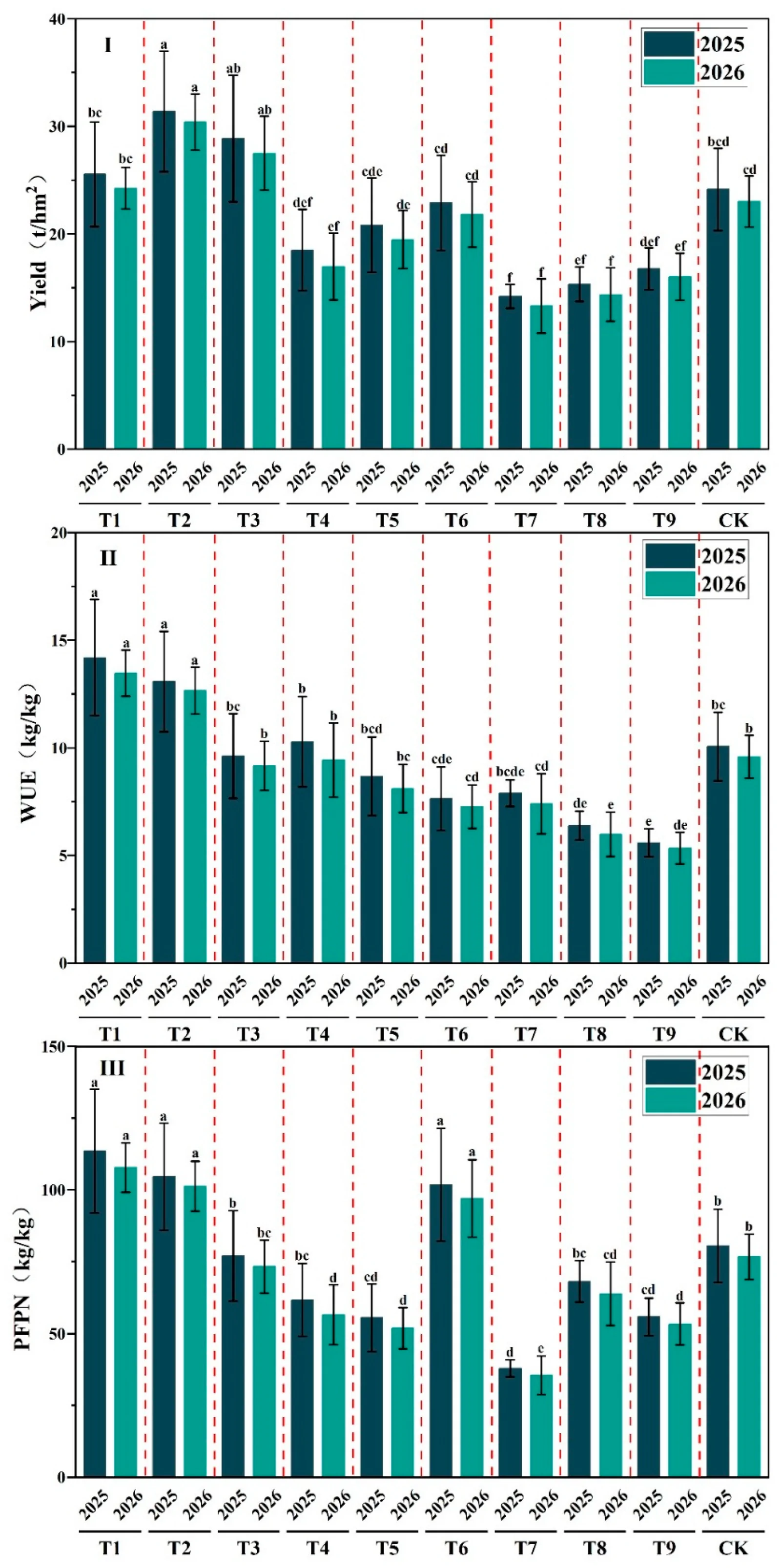
Variations in yield, water use efficiency (WUE), and partial factor productivity of applied nitrogen (PFPN) under different water–nitrogen–salinity treatments during the 2025 and 2026 growing seasons. Different letters (a–e) indicate statistically significant differences between groups. Groups with the same letter are not significantly different, while groups with different letters show significant differences. (I) yield,; (II) WUE; (III) PFPN.

Water–nitrogen–salinity treatments significantly affected crop yield and water and nitrogen use efficiencies, with considerable differences observed among treatments (Figure 6). Overall, similar variation patterns were observed for all indicators across the two experimental years, indicating consistent responses of pigment pepper production under different salinity environments and nitrogen application levels. With increasing irrigation water salinity, yield, WUE, and PFPN generally showed decreasing trends, whereas appropriate irrigation and optimized nitrogen application alleviated the negative effects of salinity and improved production performance.

Significant differences in yield were observed among different water–nitrogen–salinity treatments (Figure 6I). In 2025, yields ranged from 14.21 to 31.46 t ha^−1^, while in 2026 they ranged from 13.52 to 30.34 t ha^−1^. Similar response patterns were observed in both years. Treatment T2 produced the highest yield, reaching 31.46 and 30.34 t ha^−1^ in 2025 and 2026, respectively, whereas T7 showed the lowest yield, with only 14.21 and 13.52 t ha^−1^, respectively. Under low-salinity irrigation conditions (A1), yields were generally higher than those under medium- and high-salinity conditions (A2 and A3), indicating that reducing irrigation water salinity favored higher production levels. Under low-salinity conditions, yield initially increased and then declined with increasing irrigation amount and nitrogen application rate. Compared with the freshwater control (CK), T2 achieved higher yields under the same irrigation amount and nitrogen application rate, with yield increases of 29.89% (2025) and 32.21% (2026), respectively. Since the only difference between CK and T2 was irrigation water salinity, the improved yield performance of T2 suggests that mild salinity stress did not inhibit crop productivity but may have induced adaptive physiological responses.

Water use efficiency (WUE), reflecting the yield production capacity per unit water input, varied significantly among treatments (Figure 6II). Across the two experimental years, WUE ranged from 5.5 to 14.2 kg kg^−1^ and from 5.2 to 13.5 kg kg^−1^, respectively. Under low-salinity conditions, most treatments exhibited relatively higher WUE values, with T1 and T2 showing better performance. As irrigation water salinity increased, WUE gradually decreased, and all treatments under high salinity (A3) showed lower WUE values than those under low salinity. Irrigation amount had a significant influence on WUE. Under the same salinity level, moderately reduced irrigation input increased water productivity, whereas excessive irrigation did not further improve WUE. Notably, yield and WUE did not show completely consistent variation patterns. Some treatments exhibited relatively high WUE but lower yield levels, whereas T2 simultaneously maintained high yield and favorable WUE, indicating a better balance between production and water use efficiency.

The partial factor productivity of applied nitrogen (PFPN) further reflected variations in nitrogen use efficiency under different water–nitrogen–salinity combinations (Figure 6III). During the two experimental years, PFPN ranged from 35 to 120 kg kg^−1^ and from 30 to 115 kg kg^−1^, respectively. Overall, PFPN showed a decreasing trend with increasing nitrogen application rates. Low-nitrogen treatments generally exhibited higher PFPN, whereas high nitrogen inputs increased nitrogen supply but resulted in smaller yield increments per unit of nitrogen applied, thereby reducing PFPN. Under different salinity conditions, PFPN was generally higher under low-salinity irrigation than under high-salinity irrigation, suggesting that increased salinity reduced nitrogen productivity. Significant differences in PFPN were also observed among water and nitrogen combinations. Under low-salinity conditions, moderate nitrogen application maintained relatively high PFPN, whereas excessive nitrogen input reduced nitrogen use efficiency. Under high-salinity conditions, increasing nitrogen application could not completely compensate for the reduction in nitrogen productivity caused by salinity stress.

**Figure 7:**
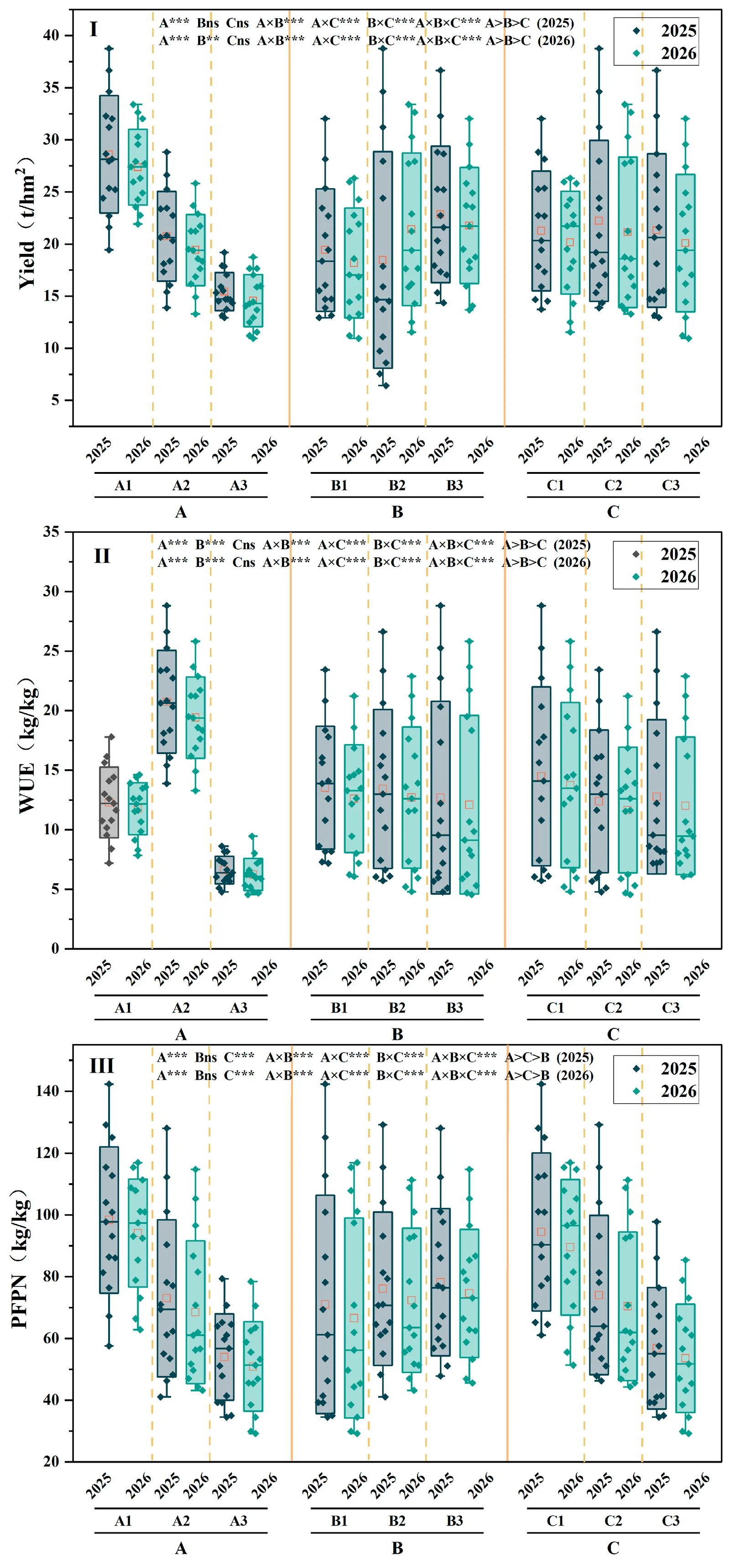
Effects of irrigation amount, irrigation water salinity, and nitrogen application rate on yield, water use efficiency (WUE), and partial factor productivity of applied nitrogen (PFPN). (**I**) yield; (**II**) WUE; (**III**) PFPN. (The box represents the interquartile range (IQR), the horizontal line inside the box indicates the median value, and the whiskers represent the range of data distribution. Points beyond the whiskers indicate outliers). Significance levels: * (*p* < 0.05), ** (*p* < 0.01), *** (*p* < 0.001), ns (*p* ≥ 0.05).

As shown in Figure 7, factor analysis revealed that different evaluation indicators exhibited distinct responses to water–nitrogen–salinity factors. Yield was mainly influenced by irrigation water salinity and irrigation amount, whereas the effect of nitrogen application rate was relatively weaker. WUE showed a stronger response to irrigation amount, indicating that water input level was an important factor regulating water productivity. PFPN was primarily affected by nitrogen application rate, demonstrating that nitrogen input determined nitrogen productivity.

Considering yield, WUE, and PFPN together, T2 exhibited the highest overall production performance across both experimental years. This treatment achieved a favorable balance between yield formation and resource use efficiency by maintaining high yield while sustaining relatively high water and nitrogen utilization efficiencies.

### 3.3. Effects of Water–Nitrogen–Salinity Interactions on Fruit Quality Traits of Pigment Pepper

**Figure 8:**
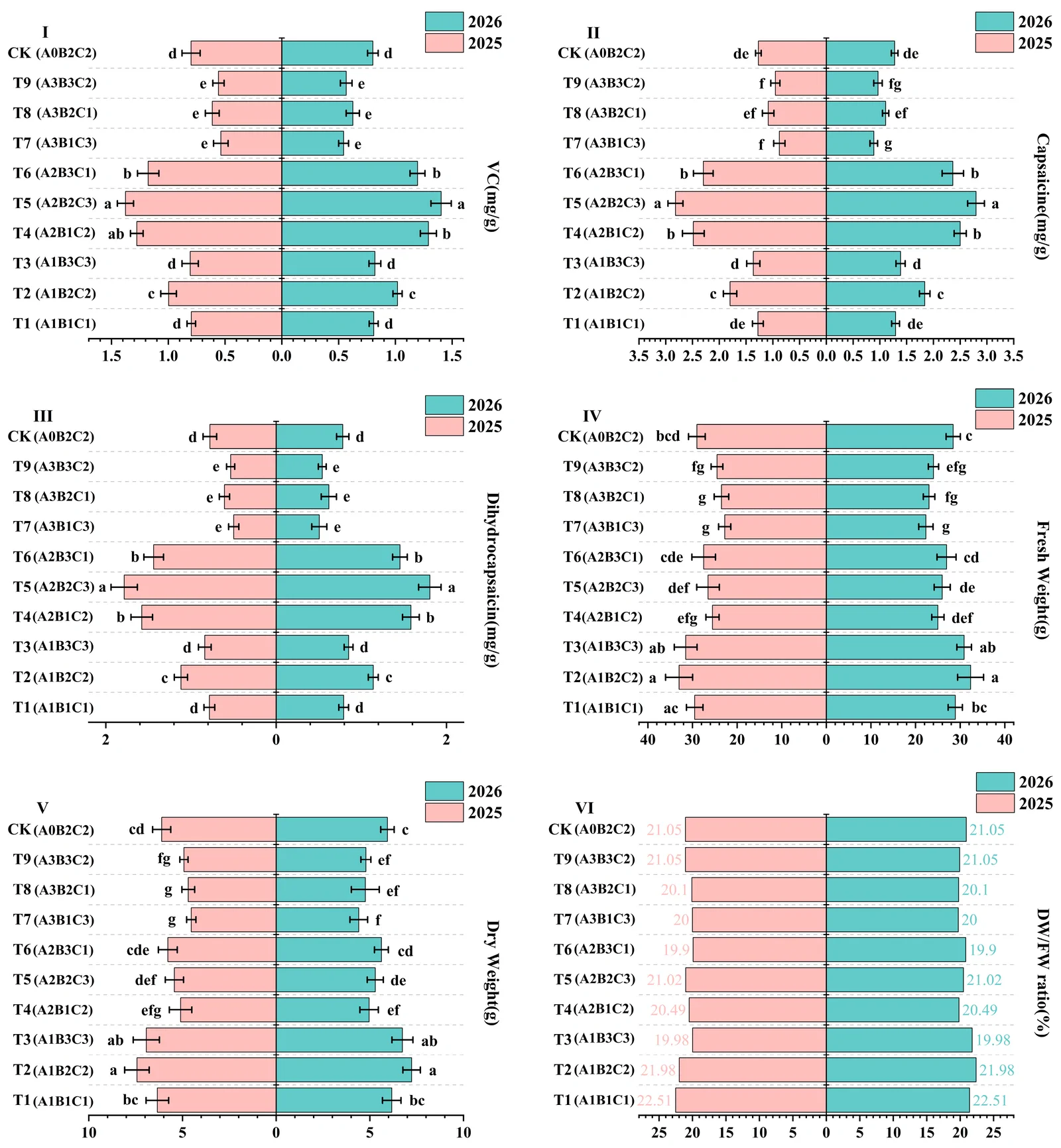
Variations in fruit quality-related traits of pigment pepper under different water–nitrogen–salinity treatments during the 2025 and 2026 growing seasons: (**I**) vitamin C content; (**II**) capsaicin content; (**III**) dihydrocapsaicin content; (**IV**) fresh weight; (**V**) dry weight; (**VI**) dry-to-fresh weight ratio. Error bars represent the standard deviation (SD) of the means. Different letters indicate significant differences among treatments according to the statistical analysis (*p* < 0.05). Treatments sharing the same letter are not significantly different.

Water–nitrogen–salinity treatments significantly affected fruit quality, formation of pigment pepper, and distinct differences in fruit biomass accumulation and quality-related traits were observed among different combinations of irrigation water salinity, irrigation amount, and nitrogen application rate (Figure 8). Overall, similar response patterns were observed between the two experimental years. Different water–nitrogen–salinity combinations significantly influenced fruit fresh weight, dry matter accumulation, and capsaicinoid contents. Appropriate water and nitrogen supply promoted fruit biomass accumulation, whereas moderate water and salinity stress showed different effects on the accumulation of capsaicinoids.

Fruit fresh weight and dry weight were significantly affected by different water–nitrogen–salinity treatments (Figure 8IV,V). During both experimental years, considerable differences were observed among treatments in terms of fruit fresh weight and dry weight. Overall, under low-salinity irrigation conditions (A1), fruit fresh weight and dry weight were generally higher than those under medium- and high-salinity conditions, indicating that a lower salinity environment favored fruit biomass accumulation. Treatment T2 (A1B2C2) exhibited relatively high fruit fresh weight and maintained favorable fruit growth performance in both years, whereas fruit fresh weight was substantially reduced under high-salinity conditions (A3). Treatments T7, T8, and T9 showed relatively lower fresh and dry weights, suggesting that high salinity limited fruit dry matter accumulation.

Under the same irrigation water salinity level, irrigation amount and nitrogen application rate showed clear regulatory effects on fruit biomass accumulation. Under low-salinity conditions, fruit fresh weight and dry weight initially increased and subsequently decreased with increasing irrigation amount and nitrogen input. Appropriate water and nitrogen combinations resulted in higher fruit biomass, whereas excessive inputs did not further enhance dry matter accumulation.

The dry-to-fresh weight ratio (DW/FW ratio), representing the proportion of dry matter accumulation in fruits, showed relatively small variations among treatments (Figure 8VI). Across the two years, DW/FW ratios remained within a relatively narrow range, indicating that water–nitrogen–salinity treatments primarily affected total fruit biomass accumulation, while their effects on fruit water proportion were comparatively limited.

Different water–nitrogen–salinity treatments significantly affected fruit vitamin C content (Figure 8I). Across the two experimental years, vitamin C content differed significantly among treatments. Under low-salinity irrigation conditions, vitamin C contents were generally higher, with T2 and T3 showing relatively better performance. With increasing irrigation water salinity, fruit vitamin C content generally declined, and its accumulation was reduced under high-salinity conditions. Under different water and nitrogen combinations, moderate increases in irrigation and nitrogen supply enhanced vitamin C content. For example, under low-salinity conditions, T3 (A1B3C3) exhibited a relatively high vitamin C content, indicating that higher water and nitrogen availability favored nutritional quality formation. However, under medium- and high-salinity conditions, the positive effects of increased water and nitrogen inputs on vitamin C accumulation were weakened.

Capsaicin and dihydrocapsaicin are important secondary metabolites determining the pungency characteristics of pepper fruits, and their accumulation showed distinct responses to water–nitrogen–salinity conditions (Figure 8II,III). Significant differences in capsaicin content were observed among treatments. Across the two experimental years, capsaicin content was generally higher under low-salinity conditions, with T3 exhibiting relatively high capsaicin accumulation, whereas treatments T5 and T6 showed relatively lower values. With increasing irrigation water salinity, capsaicin content showed a decreasing tendency, although different water and nitrogen combinations modified this response. Dihydrocapsaicin exhibited a similar variation pattern to capsaicin. Under low-salinity conditions, appropriate increases in water and nitrogen supply promoted dihydrocapsaicin accumulation, with T3 showing relatively high content. In contrast, dihydrocapsaicin contents generally decreased under high-salinity conditions.

**Figure 9:**
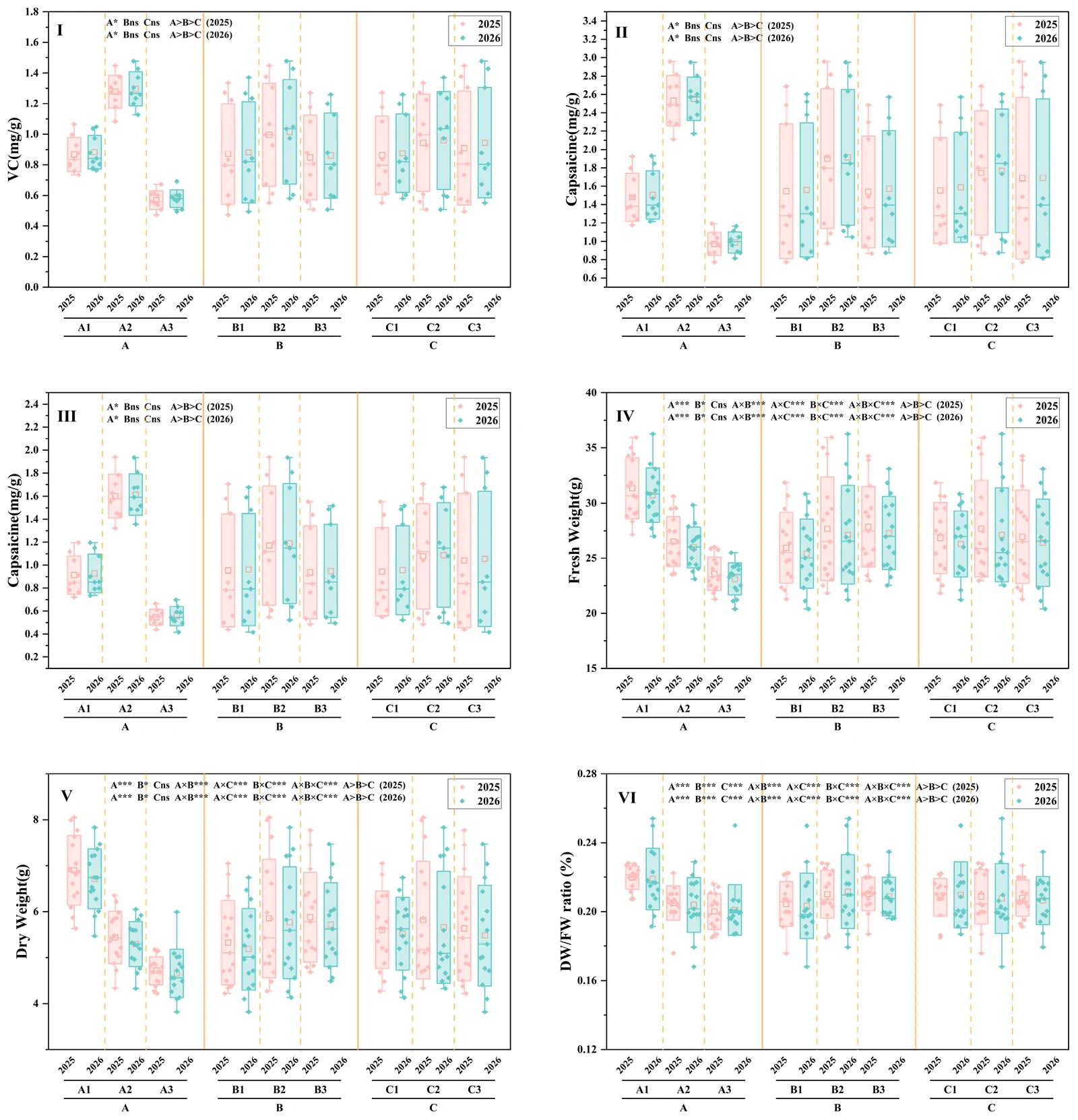
Effects of irrigation amount, irrigation water salinity, and nitrogen application rate on fruit quality-related traits of pigment pepper: (**I**) vitamin C (VC); (**II**) capsaicin; (**III**) dihydrocapsaicin; (**IV**) fresh weight; (**V**) dry weight; (**VI**) dry-to-fresh weight ratio. (The box represents the interquartile range (IQR), the horizontal line inside the box indicates the median value, and the whiskers represent the range of data distribution.Points beyond the whisk-ers indicate outliers). Significance levels: * (*p* < 0.05), ** (*p* < 0.01), *** (*p* < 0.001), ns (*p* ≥ 0.05).

As shown in Figure 9, factor effect analysis revealed different response patterns of fruit quality indicators to irrigation water salinity, irrigation amount, and nitrogen application rate. Fruit fresh weight and dry weight were mainly affected by the combined effects of salinity and water–nitrogen supply, with increasing salinity imposing clear limitations on fruit biomass formation. Vitamin C and capsaicinoids exhibited more complex response patterns, suggesting that the combined effects of water, nitrogen, and salinity played important roles in regulating their accumulation.

Overall, under low-salinity irrigation conditions, appropriate increases in irrigation amount and nitrogen input promoted fruit growth and quality development. Among all treatments, T2 maintained relatively high fruit biomass while exhibiting favorable quality-related traits, suggesting that coordinated water–nitrogen–salinity management contributed to achieving high-yield and high-quality production of pigment pepper.

### 3.4. Comprehensive Evaluation of Pigment Peppers

#### 3.4.1. Construction of a Comprehensive Evaluation Model for Pigment Pepper

The selected evaluation indicators were designed to comprehensively assess crop productivity, resource use efficiency, physiological responses, and fruit quality. Yield was considered the primary agronomic indicator because it represents the final production output and integrates the overall effects of crop management practices. Water use efficiency (WUE) was included to evaluate the efficiency of converting irrigation water into crop yield, which is particularly important in arid and water-limited regions with scarce freshwater resources. Partial factor productivity of nitrogen (PFPN) was selected as an indicator of nitrogen use efficiency, reflecting the ability of crops to transform nitrogen inputs into economic yield.

For physiological responses, the chlorophyll index (CHL), flavonoid index (FLAV), and nitrogen balance index (NBI) were incorporated because these parameters provide valuable information on photosynthetic potential, plant stress responses, and nitrogen nutritional status. These physiological traits contribute to a more comprehensive understanding of crop performance under different management conditions.

Fruit quality parameters were also included because the production value of pigment peppers depends not only on yield performance but also on quality attributes closely associated with industrial processing and commercial applications. Therefore, integrating yield, resource use efficiency, physiological characteristics, and fruit quality indicators provides a comprehensive framework for evaluating the overall performance of different production strategies.

Based on the analytic hierarchy process (AHP), a comprehensive evaluation system for pigment pepper production was established. The evaluation framework consisted of four criterion layers, including growth status, yield formation, production efficiency, and fruit quality, with a total of 12 evaluation indicators. The hierarchical structure of the evaluation system enabled the relative importance of different indicators to be quantified and provided a systematic approach for assessing the overall effects of different management strategies (Table 3).

According to the AHP-based evaluation system, the selected indicators collectively reflected the overall production performance of pigment pepper under water–nitrogen–salinity interaction conditions from the perspectives of leaf growth status, production capacity, and fruit quality. This evaluation system transformed the assessment based on individual traits into a multi-indicator comprehensive evaluation, thereby effectively reducing the evaluation bias caused by variations in single traits.

**Figure 10:**
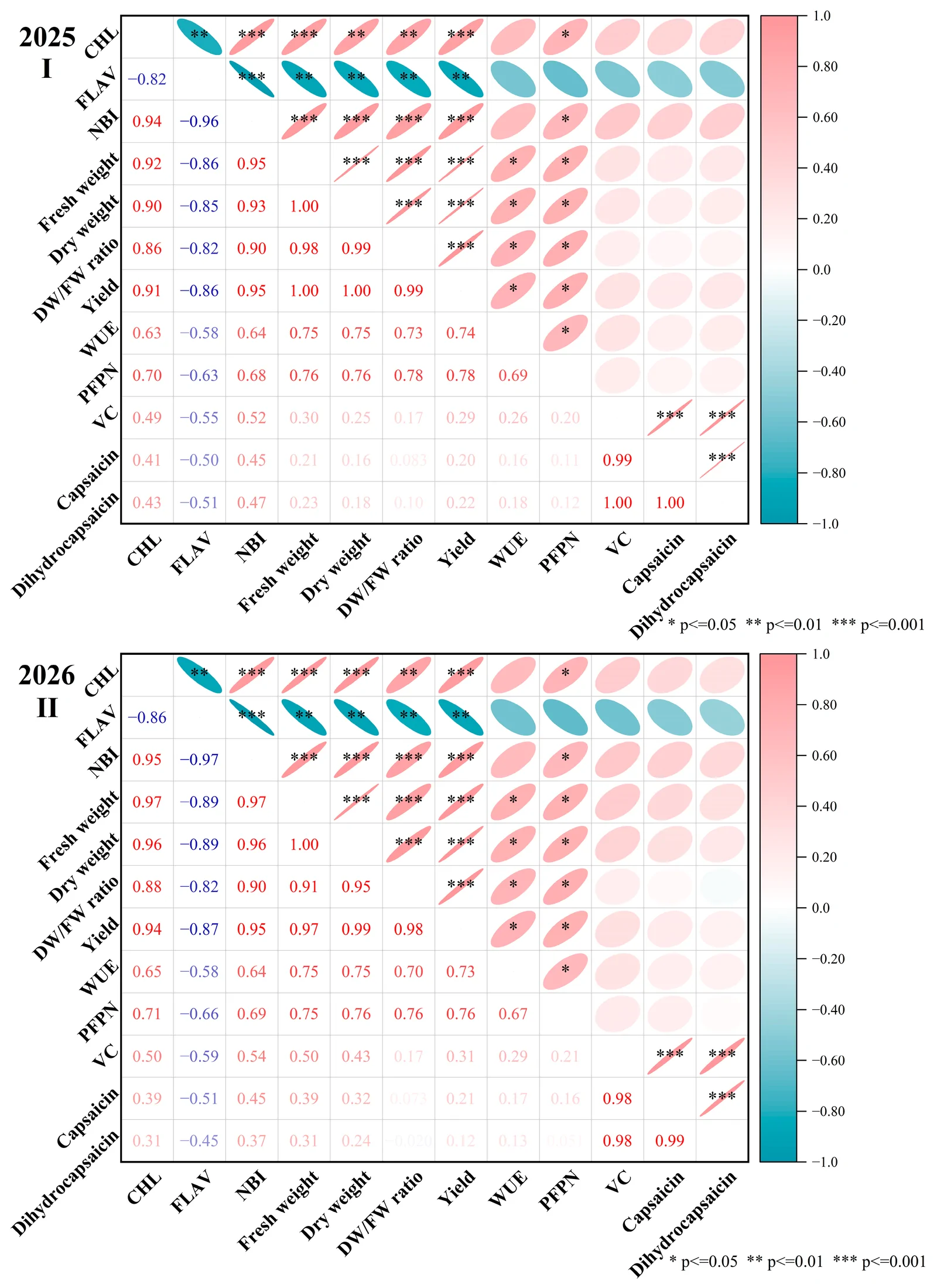
Correlation analysis among different evaluation indicators of pigment pepper in two experimental years: (**I**) 2025; (**II**) 2026. Red ellipses indicate positive correlations and blue ellipses indicate negative correlations. The shape/size of the ellipses reflects the strength of the correlation, with more elongated ellipses indicating stronger correlations. Asterisks indicate significance levels: * *p* < 0.05, ** *p* < 0.01, *** *p* < 0.001.

Further correlation analysis was conducted to elucidate the relationships among different growth-related indicators, yield-related traits, and quality-related parameters (Figure 10).

The correlation analysis revealed significant synergistic or antagonistic relationships among different indicators, with high consistency observed between the two experimental years.

Among the growth status indicators, CHL and NBI showed significant positive correlations with fruit biomass and yield. In 2025, the correlation coefficients of CHL with fruit fresh weight, fruit dry weight, and yield were 0.92, 0.90, and 0.91, respectively. These values further increased to 0.97, 0.96, and 0.94 in 2026, indicating that leaf photosynthetic status and nitrogen nutritional level were closely associated with yield formation. In contrast, FLAV exhibited significant negative correlations with other growth and production-related indicators. In both years, FLAV was negatively correlated with CHL, NBI, and yield, with correlation coefficients of −0.82 and −0.86 with CHL and −0.86 and −0.87 with yield, respectively. These results suggested that different leaf metabolic indicators exhibited distinct response patterns to variations in water–nitrogen–salinity conditions.

The fruit growth-related indicators exhibited strong consistency. Fruit fresh weight, fruit dry weight, dry matter-to-fresh weight ratio, and yield were all significantly positively correlated. Among these relationships, the correlation coefficients between fruit dry weight and yield reached 0.99 in both years, while those between fruit fresh weight and yield reached 1.00 and 0.97, respectively. This indicated that fruit dry matter accumulation was a key determinant of final yield formation.

Resource use efficiency indicators also showed positive relationships with yield. The correlation coefficients between WUE and yield were 0.74 and 0.73, while those between PFPN and yield were 0.69 and 0.67 in 2025 and 2026, respectively, indicating that improved resource use efficiency contributed to enhanced production performance. However, WUE and PFPN showed relatively weak correlations with several fruit quality indicators, suggesting a certain degree of independence between resource efficiency and quality formation.

Fruit quality indicators exhibited strong synergistic relationships. Vitamin C (VC), capsaicin, and dihydrocapsaicin contents were significantly positively correlated with each other. In particular, the correlation coefficients between capsaicin and dihydrocapsaicin reached 0.99 and 0.98 in the two years, respectively, indicating a synchronous accumulation pattern of capsaicinoids.

Overall, the correlation analysis demonstrated a high degree of stability in the relationships among evaluation indicators across the two experimental years. CHL, NBI, fruit dry weight, and yield represented the core indicators closely associated with production performance, whereas capsaicin and dihydrocapsaicin primarily reflected fruit quality characteristics. The strong correlations among several indicators suggest that some traits may capture overlapping physiological responses; therefore, these indicators were interpreted as complementary representations of plant performance rather than completely independent variables.

#### 3.4.2. Multi-Objective Decision-Making and Evaluation Based on a Cloud Model-Improved TOPSIS Method

Significant interannual variations were observed among different evaluation indicators. The subjective weights reflected the priority assigned to yield and resource use efficiency based on production experience, whereas the objective weights were derived from the variability characteristics of experimental data. A game theory-based combination weighting method was employed to balance these two aspects, ensuring the stability of indicator rankings across different years and environmental conditions while improving regional applicability. During the integration of subjective and objective weights, yield and other process-related indicators were jointly constrained based on their respective weighting characteristics. This approach not only maintained the importance of economic indicators but also avoided excessive concentration of weights and potential double counting among correlated indicators.

According to the subjective weighting results (Table 4), yield was identified as the primary evaluation indicator during the growing season, with a weight of 0.400, whereas fruit fresh weight exhibited the lowest weight (0.014). However, substantial interannual variations were observed in the objective weights derived from the entropy weight method. The dry matter-to-fresh weight ratio was identified as the most influential indicator in 2023, with a weight of 0.1205. In 2024, this indicator exhibited a slightly higher weight of 0.1238, maintaining its dominant contribution among the objective indicators.

Based on the integrated weights obtained by combining subjective and objective weights through the game theory-based weighting method, yield consistently maintained the highest importance across years, with identical weights of 0.2812 in both 2023 and 2024. By integrating expert knowledge and data-driven information, this approach effectively balanced subjective preferences and objective variability, thereby enhancing the stability and scientific reliability of the comprehensive evaluation results. Meanwhile, the integrated weight of the nitrogen balance index (NBI) remained relatively low in both growing seasons (0.0478), indicating its comparatively limited contribution within the overall evaluation framework.

**Figure 11:**
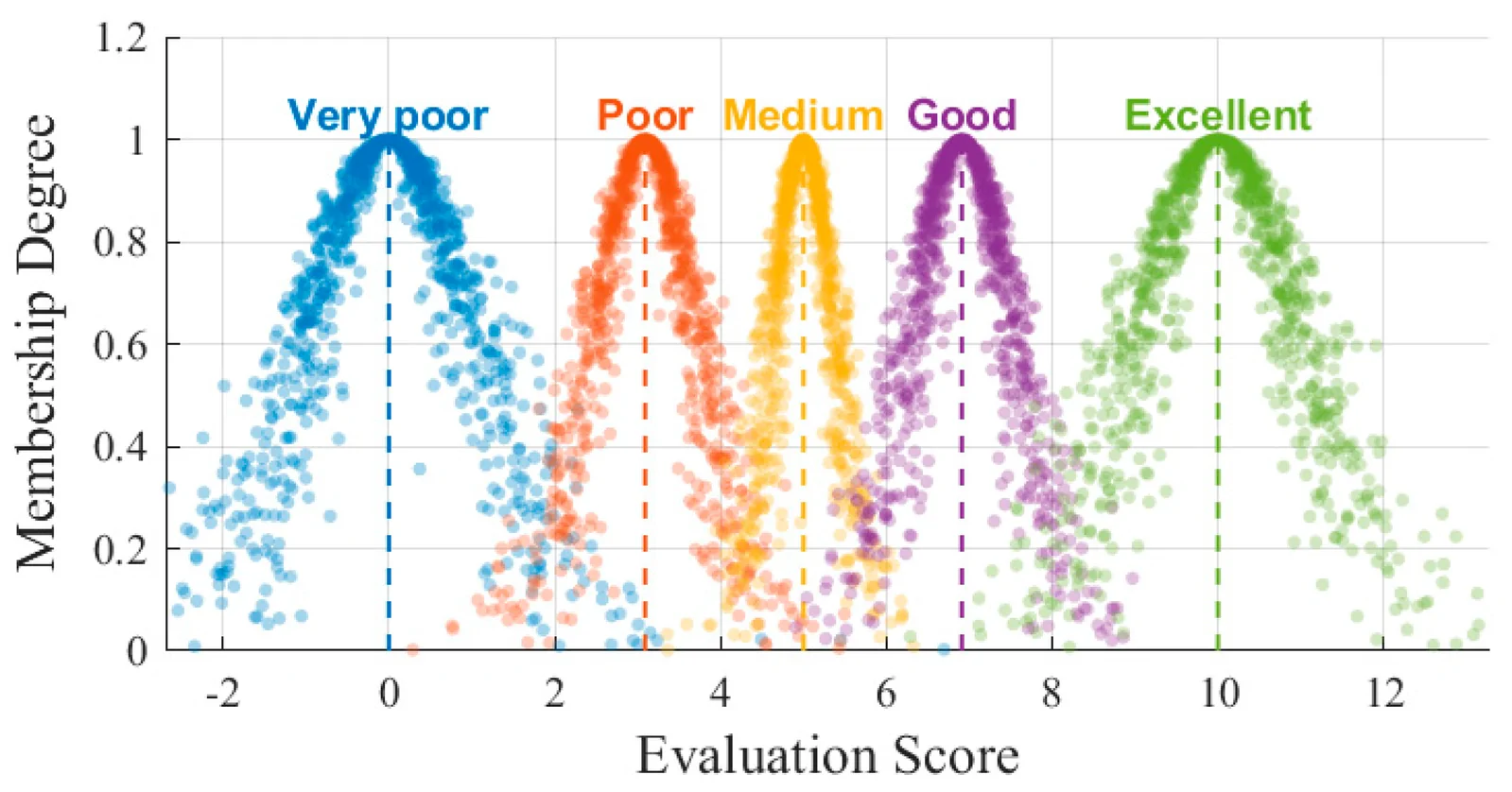
Membership degree standard cloud chart.

A standard evaluation cloud model was established based on the criteria of high yield, improved fruit quality, reduced water and nitrogen inputs, and optimal growth status by integrating the comprehensive indicator values of each treatment (Figure 11).

The indicator weights obtained from the game theory-based combination weighting method were integrated with the cloud model to quantify linguistic evaluation information, and the closeness coefficients of different treatments were calculated using the TOPSIS approach. As shown in Table 5, treatment T2 achieved the highest comprehensive evaluation score in both experimental years, suggesting that this treatment provided an effective balance among water, fertilizer, and salinity regulation. The rankings of most treatments remained relatively stable across years. In contrast, treatments T7, T8, and T9 exhibited consistently lower scores and were positioned near the bottom of the ranking list in both years.

## 4. Discussion

### 4.1. Effects of Water–Nitrogen–Salinity Interactions on Growth and Physiological Adaptation Mechanisms of Pigment Pepper

Water–nitrogen–salinity interactions regulate leaf growth and physiological metabolic processes of pigment pepper by altering root-zone water and salinity conditions as well as nutrient availability. In the present study, different combinations of brackish water salinity, irrigation amount, and nitrogen application rate significantly affected leaf chlorophyll content (CHL), flavonoid content (FLAV), and nitrogen balance index (NBI), with high consistency observed between the two experimental years. These results indicate that water–nitrogen–salinity combinations exerted stable regulatory effects on the physiological status of pigment pepper. The results showed that low-salinity irrigation (1 g L^−1^) combined with appropriate irrigation and nitrogen supply maintained relatively higher CHL and NBI values, whereas high-salinity conditions (4 g L^−1^) resulted in reductions in photosynthetic pigments and nitrogen nutritional status.

Salt stress initially restricts plant water uptake by reducing the water potential in the root zone, leading to physiological drought. Meanwhile, excessive accumulation of Na^+^ and Cl^−^ disrupts ionic homeostasis, further affecting chloroplast structural stability and photosynthetic processes. Therefore, the decline in chlorophyll content under high-salinity irrigation reflects the combined effects of osmotic limitation and ionic toxicity on photosynthetic regulation. Reduced water availability at the root–soil interface restricts stomatal opening and CO_2_ diffusion, while excessive Na^+^ and Cl^−^ accumulation may impair chloroplast stability and interfere with pigment synthesis, ultimately reducing photosynthetic carbon assimilation. In this study, CHL significantly decreased under treatment A3, which was consistent with previous studies demonstrating that salt stress inhibits chlorophyll biosynthesis and causes damage to photosynthetic systems [24,25,26,27,28].

However, moderate salinity did not completely inhibit plant growth but instead induced a certain degree of stress adaptation in pigment pepper. Previous studies have shown that mild-to-moderate salinity conditions can promote the accumulation of osmotic adjustment substances and antioxidant compounds, thereby enhancing plant tolerance to adverse environments [29,30,31]. In addition to physiological and biochemical adjustments, plant-associated microorganisms, particularly endophytic fungi, have also been reported to contribute to plant adaptation to saline environments by regulating stress responses and improving salt tolerance [32]. In the present study, FLAV increased with increasing salinity levels, suggesting that pigment pepper may alleviate oxidative damage caused by salt stress through enhanced flavonoid-related secondary metabolism. As important non-enzymatic antioxidants, flavonoids can scavenge reactive oxygen species (ROS) and maintain cellular membrane stability; therefore, their accumulation is considered a key regulatory mechanism underlying plant adaptation to salt stress [33].

Furthermore, irrigation amount and nitrogen supply modulated plant physiological responses under salt stress conditions. In this study, increasing irrigation amount under the same salinity level enhanced CHL and NBI values, indicating that appropriate increases in water input improved root-zone salinity conditions by promoting salt leaching and reducing osmotic stress, thereby facilitating water and nitrogen uptake by plants. However, excessive irrigation did not continuously enhance leaf physiological indicators, suggesting that simply increasing irrigation inputs cannot achieve optimal physiological benefits in arid agricultural systems. Instead, irrigation management should be coordinated with salinity levels and nutrient availability to achieve synergistic regulation.

Nitrogen supply mainly influences leaf nitrogen nutritional status and photosynthetic capacity; however, its effects are constrained by the prevailing water and salinity conditions. In the present study, the effects of nitrogen application were weaker than those of salinity and irrigation amount for several physiological indicators, which is consistent with previous studies showing that irrigation management and salinity conditions exert stronger influences on pepper growth than nitrogen supply alone [34,35,36,37]. These findings suggest that in saline agricultural regions, improving root-zone water and salinity conditions may be more effective for promoting crop growth than simply increasing nitrogen inputs.

### 4.2. Regulatory Mechanisms of Water–Nitrogen–Salinity Interactions on Yield Formation and Water and Nitrogen Use Efficiency

Yield formation represents the integrated response of crops to variations in water, nutrient, and salinity conditions. In this study, different water–nitrogen–salinity combinations significantly affected yield, water use efficiency (WUE), and partial factor productivity of nitrogen fertilizer (PFPN) of pigment pepper, with consistent response patterns observed across the two experimental years. Among all treatments, T2 (A1B2C2, corresponding to a salinity level of 1 g L^−1^, irrigation amount of 2400 m^3^ ha^−1^, and nitrogen application rate of 300 kg ha^−1^) achieved a relatively high yield while maintaining superior resource use efficiency.

Interestingly, T2 exhibited higher yield performance than the freshwater control (CK), although both treatments received identical irrigation amounts and nitrogen inputs. Since the main difference between these two treatments was irrigation water salinity, this result indicates that low-level salinity did not necessarily impose a detrimental effect on crop production under appropriate water and nitrogen management. Mild salinity may stimulate adaptive physiological regulation, including osmotic adjustment and improved water-use coordination, allowing plants to maintain relatively stable photosynthetic activity and nutrient acquisition. In this study, the favorable CHL and NBI performance under T2 conditions provided physiological support for sustained carbon assimilation and biomass accumulation, ultimately contributing to enhanced yield formation.

Salinity level was identified as a major limiting factor affecting yield formation. High-salinity irrigation promoted salt accumulation in the root zone, reduced root water uptake capacity, and restricted nutrient transport and photosynthetic assimilate production, ultimately resulting in yield reduction [38]. In this study, yield under treatment A3 was significantly lower than that under A1, indicating that a salinity level of 4 g L^−1^ exceeded the suitable salinity tolerance range of pigment pepper.

However, appropriate water and nitrogen supply alleviated the adverse effects of salt stress. Increasing the irrigation amount not only improved soil water availability but also facilitated salt migration to deeper soil layers, thereby reducing salt accumulation in the root zone. Adequate nitrogen supply enhanced leaf nitrogen status and supported chlorophyll synthesis, thereby maintaining photosynthetic enzyme activity and carbon assimilation capacity. However, nitrogen utilization efficiency depended strongly on soil water and salinity conditions, because excessive salt accumulation could limit root uptake and reduce the conversion efficiency of applied nitrogen into biomass [39]. Therefore, under low-salinity conditions, moderate increases in irrigation and nitrogen inputs promoted yield formation. However, excessive nitrogen application did not further increase yield but instead reduced PFPN, indicating the existence of a nitrogen input threshold [40].

WUE reflects the relationship between crop yield formation and water consumption. The present study showed that WUE was generally higher under low-salinity conditions, whereas high salinity reduced crop productivity per unit of water consumed. This reduction was mainly attributed to the inhibition of transpiration efficiency and photosynthetic carbon assimilation caused by salt stress, which limited the conversion of additional water inputs into economic yield. Meanwhile, some low-irrigation treatments exhibited relatively high WUE but lower yield levels, indicating that maximizing water efficiency alone may result in yield penalties. Therefore, agricultural water management in arid regions should seek an appropriate balance between yield production and water use efficiency rather than focusing solely on improving WUE [41].

The PFPN results further demonstrated that nitrogen use efficiency was jointly affected by salinity conditions and nitrogen application levels. PFPN generally decreased with increasing nitrogen input, which was mainly due to the gradual reduction in yield increments with additional nitrogen application [42]. These results indicate that under brackish water irrigation conditions, increasing nitrogen inputs cannot fully compensate for production limitations caused by salt stress. Therefore, optimizing root-zone water and salinity conditions is more important than simply increasing fertilizer inputs for improving crop productivity.

Under low-salinity irrigation conditions, optimized water and nitrogen management alleviated salt stress and improved yield formation and resource use efficiency. Therefore, the T2 treatment (1 g L^−1^ salinity combined with appropriate irrigation and nitrogen input) achieved the best comprehensive production performance under the experimental conditions. Although low-salinity brackish water (1 g L^−1^) was optimal for comprehensive production performance, moderate salinity (2 g L^−1^) showed potential benefits for improving certain fruit quality attributes, indicating that salinity regulation should consider the balance between yield formation and quality improvement. This suggests that brackish water management should not only focus on minimizing salinity stress but also consider the potential quality-enhancing effects of moderate salinity levels.

### 4.3. Effects of Water–Nitrogen–Salinity Interactions on Fruit Quality Formation of Pigment Pepper

In addition to yield formation, fruit quality is an important indicator for evaluating the production benefits and economic value of pigment pepper. In this study, water–nitrogen–salinity interactions significantly affected fruit biomass accumulation and quality-related compound formation. Under different combinations of irrigation water salinity, irrigation amount, and nitrogen application rate, significant variations were observed in fruit fresh weight, fruit dry weight, vitamin C content, and capsaicinoid accumulation. Overall, low-salinity conditions were beneficial for maintaining higher fruit biomass, whereas moderate salinity promoted the accumulation of certain secondary metabolites, indicating the dual regulatory effects of water and salinity management on both yield formation and quality improvement of pigment pepper.

Salinity exhibited a clear dose-dependent effect on fruit quality formation. On the one hand, excessive salinity reduced root-zone water potential, increased osmotic stress, and restricted root uptake of water and mineral nutrients. These processes subsequently reduced leaf photosynthetic capacity and carbon assimilation, limiting the supply of assimilates and ultimately inhibiting fruit dry matter accumulation. In this study, fruit fresh weight and dry weight were significantly reduced under high-salinity treatment (A3), suggesting that prolonged salt stress beyond the adaptive capacity of plants inhibited the translocation of photosynthetic products to fruits and negatively affected quality formation [43]. On the other hand, moderate salt stress can act as an environmental signal to activate plant defense-related metabolism, promoting the accumulation of phenolics, flavonoids, and capsaicinoids, thereby enhancing fruit quality characteristics [44].

This balance between growth accumulation and defense metabolism reflects a resource allocation trade-off. Under favorable conditions, assimilated carbon is preferentially allocated toward biomass production and fruit development, whereas moderate stress conditions redirect part of carbon flux toward secondary metabolism associated with defense responses. Under non-stress or mild-stress conditions, plants preferentially allocate resources toward vegetative growth and carbon assimilation. However, under moderate salinity stimulation, part of the carbon resources and energy may be redirected toward secondary metabolic pathways to enhance stress adaptation. Previous studies have demonstrated that moderate water deficit or salinity stress in Capsicum chinense and Capsicum frutescens can promote capsaicinoid biosynthesis by enhancing the activities of phenylalanine ammonia-lyase (PAL) and capsaicin synthase (CS), thereby increasing capsaicinoid accumulation [45,46,47,48,49,50]. In the present study, FLAV showed strong correlations with quality-related indicators, further confirming the important regulatory role of secondary metabolism in pigment pepper quality formation.

However, stress-induced quality enhancement depends on appropriate water and nitrogen supply conditions. Adequate irrigation can improve root-zone water and salinity conditions, enhance plant water status, and promote photosynthetic carbon supply, thereby providing the foundation for fruit growth and quality compound synthesis [51]. However, excessive irrigation may weaken the intensity of mild stress signals in the root zone and reduce the induction of plant defense metabolism, thereby limiting the accumulation of certain secondary metabolites. Therefore, increasing water input alone cannot continuously improve fruit quality; instead, irrigation management should be coordinated with salinity levels and nitrogen supply to achieve optimal regulation.

Correlation analysis further demonstrated that chlorophyll content (CHL) and nitrogen balance index (NBI) were closely associated with yield formation, whereas FLAV exhibited stronger relationships with quality-related indicators. These results indicate the existence of a coordinated regulatory mechanism between photosynthetic nitrogen metabolism and secondary metabolism during pigment pepper production. An appropriate water–fertilizer–salinity combination can simultaneously maintain high photosynthetic productivity and moderate stress-induced metabolic activity, providing an essential basis for achieving high yield and superior quality.

In this study, treatment T2 (A1B2C2) maintained relatively high fruit fresh weight and dry matter accumulation while exhibiting favorable quality characteristics, indicating that low-salinity irrigation combined with moderate irrigation and appropriate nitrogen input achieved coordinated optimization between yield formation and quality improvement. Therefore, water–nitrogen–salinity management strategies for pigment pepper production in arid regions of Xinjiang should not solely focus on maximizing yield but should also consider resource use efficiency and quality benefits. Precise regulation of root-zone water and salinity conditions is essential for achieving efficient and sustainable crop production.

### 4.4. Optimization of Water–Nitrogen–Salinity Interactions and Comprehensive Production Performance Evaluation

Due to the significant interactions among water, fertilizer, and salinity factors, the performance of different management strategies cannot be accurately evaluated using a single indicator alone. The strong correlations among physiological, yield-related, and quality-related indicators also indicate that several measured traits may represent common regulatory processes. For example, the close associations between CHL, NBI, biomass accumulation, and yield suggest that photosynthetic capacity and nitrogen nutritional status jointly determine production performance. However, these indicators were retained in the evaluation framework because they provide information from different biological perspectives. CHL and NBI mainly reflect leaf physiological status, whereas yield, WUE, PFPN, and fruit quality represent the final consequences of resource allocation and crop management. Therefore, integrating multiple indicators allows for a more comprehensive evaluation while avoiding reliance on a single trait. Therefore, a combined weighting method and the Cloud–TOPSIS approach were further applied in this study to comprehensively evaluate growth status, yield formation, production efficiency, and fruit quality [52]. Similar multi-objective evaluation frameworks have been increasingly applied in agricultural water and nutrient management to simultaneously balance crop productivity, resource use efficiency, and environmental sustainability [53]. By integrating CHL, FLAV, NBI, yield, WUE, PFPN, and fruit-quality-related indicators, the evaluation system provided a more comprehensive assessment of pigment pepper production performance under different water–nitrogen–salinity management strategies. Meanwhile, the combined weighting method reduced the potential bias caused by assigning equal importance to highly correlated indicators by considering both expert knowledge and data variability.

The comprehensive evaluation results showed that treatment T2 achieved the highest comprehensive closeness coefficient, indicating that this management strategy effectively coordinated growth performance, yield formation, resource use efficiency, and fruit quality through appropriate irrigation and nitrogen supply under relatively low salt input conditions.

These findings further demonstrate that brackish water utilization in arid regions of Xinjiang should not focus solely on maximizing yield but should instead shift toward a comprehensive objective of “stable yield, high resource efficiency, and improved quality”. This concept is consistent with recent studies emphasizing that integrated irrigation and nutrient optimization can enhance crop productivity while improving water and fertilizer utilization efficiency under water-limited conditions [54]. From a practical perspective, the results suggest that brackish water irrigation should not be considered only as a substitute for freshwater under water-limited conditions but as a management resource requiring precise regulation. For pigment pepper production in arid regions, low-salinity brackish water combined with moderate irrigation and a balanced nitrogen supply can maintain yield stability while improving resource use efficiency. However, excessive salt input or unnecessary nitrogen application may reduce production benefits by increasing osmotic stress and decreasing nutrient utilization efficiency.

## 5. Conclusions

This study systematically investigated the effects of brackish water salinity, irrigation amount, and nitrogen application rate on the growth performance, yield formation, water and nitrogen use efficiency, and fruit quality of pigment pepper through two years of field experiments in the arid region of Xinjiang. The response mechanisms of pigment pepper production under water–nitrogen–salinity interaction conditions were elucidated, and the following conclusions were obtained:

(1) Water–nitrogen–salinity interactions significantly regulated the physiological status of pigment pepper leaves. Increasing irrigation water salinity generally reduced leaf CHL and NBI, whereas FLAV exhibited a stress-induced accumulation pattern. Low-salinity irrigation combined with appropriate water and nitrogen management maintained higher photosynthetic capacity and nitrogen nutritional status. Among the tested treatments, T2 exhibited superior leaf growth performance during multiple growth stages.
(2) The synergistic regulation of water, fertilizer, and salinity determined yield formation and resource use efficiency of pigment pepper. High-salinity irrigation significantly reduced yield, WUE, and PFPN, whereas reduced salt input combined with optimized irrigation and nitrogen supply improved production performance. Treatment T2 (A1B2C2) achieved the highest yield in both experimental years and effectively balanced yield formation, water use efficiency, and nitrogen use efficiency.
(3) Water–nitrogen–salinity management strategies affected the quality formation process of pigment pepper. Low-salinity conditions promoted fruit biomass accumulation, whereas moderate salt stress stimulated capsaicinoid accumulation, indicating that quality improvement depended on the balance between biomass production and stress-induced metabolic regulation. Correlation analysis demonstrated that photosynthetic nitrogen metabolism was closely associated with yield formation, while flavonoid accumulation played an important role in quality development.
(4) According to the comprehensive evaluation results based on the combined weighting Cloud–TOPSIS approach, T2 was identified as the optimal water–nitrogen–salinity management strategy under the experimental conditions. This strategy achieved coordinated optimization of yield, fruit quality, and resource use efficiency through appropriate water and nitrogen regulation under reduced salt input conditions and therefore provides an effective management option for the safe utilization of brackish water in pigment pepper production in arid regions of Xinjiang.

## Figures and Tables

**Table 1:** Table of basic physical and chemical properties of soil.

Soil Depth (cm)	Granulometric Analysis (%)			EC (us·cm^−1^)		PH		Bulk Density (g·cm^−3^)	
	Clay (<0.002 mm)	Silt (0.002~0.02)	Sand (0.02~2 mm)	2025	2026	2025	2026	2025	2026
0–20	3.4	32.95	63.65	278	306	8.25	8.01	1.354	1.327
20–40	4.6	33.22	65.84	286	328	8.65	8.43	1.268	1.243
40–60	5.1	34.56	61.68	304	323	8.71	8.51	1.292	1.266
60–80	6.5	34.56	58.94	297	331	8.68	8.33	1.401	1.380

**Table 2:** L_9_(3^3^) orthogonal experimental design.

Test Number	Treatment Combination	A Salinity Levels of Brackish Water (g·L)	B Irrigation Amounts (m^3^·hm^−1^)	C Nitrogen Application Rate (kg·hm^−1^)
T1	A1B1C1	1	1800	225
T2	A1B2C2	1	2400	300
T3	A1B3C3	1	3000	375
T4	A2B1C2	2	1800	300
T5	A2B2C3	2	2400	375
T6	A2B3C1	2	3000	225
T7	A3B1C3	4	1800	375
T8	A3B2C1	4	2400	225
T9	A3B3C2	4	3000	300
	Level			
	1	1	1800	225
	2	2	2400	300
	3	4	3000	375

**Table 3:** Comprehensive evaluation system for the growth of pigment peppers based on the AHP (analytic hierarchy process).

Goal Layer	Criteria Layer	Indicator Layer
Comprehensive Evaluation of Pigment Pepper Growth A	Growth Status B1	CHL C1
		FLAV C2
		NBI C3
	Yield Formation B2	Yield C4
	Production Efficiency B3	WUE C5
		PFPN C6
	Fruit Quality B4	Fresh weight C7
		Dry weight C8
		DW/FW ratio C9
		VC C10
		Capsaicin C11
		Dihydrocapsaicin C12

**Table 4:** Indicator weights derived from AHP, entropy weight method, and game theory-based combination weighting across different years.

Parameters	AHP	Entropy Weight		Combined Weight	
		2025	2026	2025	2026
CHL	0.1078	0.0683	0.0710	0.0756	0.0755
FLAV	0.0328	0.0788	0.0739	0.0500	0.0496
NBI	0.0594	0.0596	0.0587	0.0478	0.0478
Yield	0.4000	0.0779	0.0789	0.2812	0.2812
WUE	0.1334	0.0712	0.0731	0.0909	0.0908
PFPN	0.0666	0.0558	0.0591	0.0511	0.0511
Fresh weight	0.0140	0.0757	0.0749	0.0529	0.0529
Dry weight	0.0440	0.0861	0.0857	0.0579	0.0580
DW/FW ratio	0.0220	0.1205	0.1238	0.0874	0.0879
VC	0.0300	0.0952	0.0936	0.0634	0.0633
Capsaicin	0.0600	0.1046	0.1030	0.0706	0.0706
Dihydrocapsaicin	0.0300	0.1064	0.1045	0.0713	0.0712

**Table 5:** Comprehensive evaluation results of pigment pepper growth based on Cloud–TOPSIS.

Treatment Code	2025		2026	
	Score	Ranking	Score	Ranking
T1	0.5604	3	0.5598	3
T2	0.6914	1	0.6957	1
T3	0.5915	2	0.5925	2
T4	0.458	6	0.4486	7
T5	0.5136	5	0.5092	5
T6	0.5533	4	0.5546	4
T7	0.2303	9	0.2283	9
T8	0.196	10	0.1891	10
T9	0.2328	8	0.2293	8
CK	0.4574	7	0.455	6

## Data Availability

The original contributions presented in the study are included in the article; further inquiries can be directed to the corresponding author.

## Abbreviations

The following abbreviations are used in this manuscript:

CHL	Chlorophyll
FLAV	Flavonoids
NBI	Nitrogen nutrition index
VC	Vitamin C
WUE	Water use efficiency
PFPN	Partial factor productivity of applied nitrogen
